# Peroxisomal β-oxidation acts as a sensor for intracellular fatty acids and regulates lipolysis

**DOI:** 10.1038/s42255-021-00489-2

**Published:** 2021-12-13

**Authors:** Lianggong Ding, Wenfei Sun, Miroslav Balaz, Anyuan He, Manuel Klug, Stefan Wieland, Robert Caiazzo, Violeta Raverdy, Francois Pattou, Philippe Lefebvre, Irfan J. Lodhi, Bart Staels, Markus Heim, Christian Wolfrum

**Affiliations:** 1 Institute of Food, Nutrition and Health, ETH Zürich, Schwerzenbach, Switzerland; 2 Institute of Experimental Endocrinology, Biomedical Research Center at the Slovak Academy of Sciences, Bratislava, Slovakia; 3 Department of Animal Physiology and Ethology, Faculty of Natural Sciences, Comenius University in Bratislava, Bratislava, Slovakia; 4 Division of Endocrinology, Metabolism and Lipid Research, Department of Medicine, Washington University School of Medicine, St. Louis, MO USA; 5 School of Life Sciences, Anhui Medical University, Hefei, China; 6 Hepatology, Department of Biomedicine, University Hospital and University of Basel, Basel, Switzerland; 7 University Lille, CHU Lille, Institut Pasteur Lille, Inserm, UMR1190 Translational Research in Diabetes, Lille, France; 8 University Lille, Inserm, CHU Lille, Institut Pasteur de Lille, U1011-EGID, Lille, France; 9 Division of Gastroenterology and Hepatology, Clarunis, University Center for Gastrointestinal and Liver Diseases, Basel, Switzerland

**Keywords:** Metabolic diseases, Peroxisomes, Fatty acids

## Abstract

To liberate fatty acids (FAs) from intracellular stores, lipolysis is regulated by the activity of the lipases adipose triglyceride lipase (ATGL), hormone-sensitive lipase and monoacylglycerol lipase. Excessive FA release as a result of uncontrolled lipolysis results in lipotoxicity, which can in turn promote the progression of metabolic disorders. However, whether cells can directly sense FAs to maintain cellular lipid homeostasis is unknown. Here we report a sensing mechanism for cellular FAs based on peroxisomal degradation of FAs and coupled with reactive oxygen species (ROS) production, which in turn regulates FA release by modulating lipolysis. Changes in ROS levels are sensed by PEX2, which modulates ATGL levels through post-translational ubiquitination. We demonstrate the importance of this pathway for non-alcoholic fatty liver disease progression using genetic and pharmacological approaches to alter ROS levels in vivo, which can be utilized to increase hepatic ATGL levels and ameliorate hepatic steatosis. The discovery of this peroxisomal β-oxidation-mediated feedback mechanism, which is conserved in multiple organs, couples the functions of peroxisomes and lipid droplets and might serve as a new way to manipulate lipolysis to treat metabolic disorders.

## Abstract (web summary)

Ding et al. report a mechanism that allows cells to sense cellular fatty acid levels and adjust lipolysis as needed, which involves peroxisomal degradation of fatty acids, production of reactive oxygen species and post-translational regulation of adipose triglyceride lipase.

## Main

Peroxisomes are organelles, which are present in virtually all eukaryotic cells and play an important role in numerous processes, linked to lipid metabolism^1^. While medium- and long-chain FAs are mainly oxidized in mitochondria and to a lesser degree in peroxisomes^2,3^, very-long-chain FAs (VLCFAs) are almost exclusively metabolized by β-oxidation in peroxisomes^4^, exemplified by increased plasma VLCFA levels^5^ in various peroxisome biogenesis disorders. The rate-limiting step in peroxisomal β-oxidation is dependent on acyl-CoA oxidase 1 (ACOX1), a peroxisomal matrix protein, which catalyzes desaturation of acyl-CoAs to 2-trans-enoyl-CoAs, resulting in generation of hydrogen peroxide (H_2_O_2_), which accounts for up to 35% of the total H_2_O_2_ production in mammalian tissues^6^. *ACOX1* loss of function leads to pseudoneonatal adrenoleukodystrophy, increased plasma VLCFA levels and glial degeneration^7^. Conversely, *ACOX1* gain-of-function mutations result in a progressive glial degeneration, due to enhanced oxidative stress caused by excessive H_2_O_2_ production, which can be pharmacologically attenuated by treatment with the antioxidant *N*-acetyl cysteine amide (NACA)^8^. To efficiently counteract the oxidative stress caused by elevated peroxisomal β-oxidation, peroxisomes import catalase (CAT) into the matrix, which can convert H_2_O_2_ into H_2_O and O_2_ (ref. ^9^).

To import matrix proteins into peroxisomes, a coordinated function of different peroxins (PEXs) is required. The cytosolic receptors PEX5 and PEX7 recognize and interact with peroxisome-targeting sequence-1 (PTS1) and -2 (PTS2) containing cargo proteins, complemented by docking to the peroxisomal membrane through PEX13 and PEX14 (ref. ^5^). The peroxisomal membrane proteins PEX2/10/12, three RING (Really Interesting New Gene) finger E3 ligases, ubiquitinate PEX5 to initiate PEX5 recycling into cytosol or for further degradation^5^. In addition to PEX5 ubiquitination, PEX2 also plays an important role in pexophagy regulation and only the levels of PEX2 among the PEX2–PEX10–PEX12 protein complex are strictly controlled at normal state via an unknown mechanism^10^.

Lipid droplets (LDs) are storage organelles for lipid deposition that are mainly composed of neutral lipids such as triacylglycerols (TAGs) and cholesteryl esters (CEs)^11^. In times of energy demand, LDs are depleted by TAG hydrolysis to liberate FA by either lipolysis or lipophagy, which are intricately regulated by insulin and catecholamines^12^. Lipolysis is mainly driven by the action of ATGL, hormone-sensitive lipase (HSL) and monoacylglycerol lipase (MGL), which convert TAGs in a stepwise fashion into glycerol and FAs^12–14^. The first rate-limiting enzyme ATGL mainly localizes in the adipocyte cytosol in the basal state and is recruited to the LD surface upon hormonal stimulation^15^. However, ATGL is normally tethered on the LD surface through a hydrophobic domain (HD) in non-adipocyte cells^13,15^.

It is well documented that peroxisomes and LDs are in physical contact within cells^16^. Apart from a recent report demonstrating that peroxisome–LD contacts are essential for ATGL redistribution to the LD surface upon hormone stimulation in adipocytes^17^, the molecular basis for physical interaction and functional interconnectivity of the two organelles remains largely unexplored. Here we show that peroxisomal β-oxidation functions as a sensor of intracellular FAs and regulates lipolysis via ATGL ubiquitination.

## Results

### PEX2/10/12 modulate lipolysis by regulating ATGL protein

**Fig. 1:**
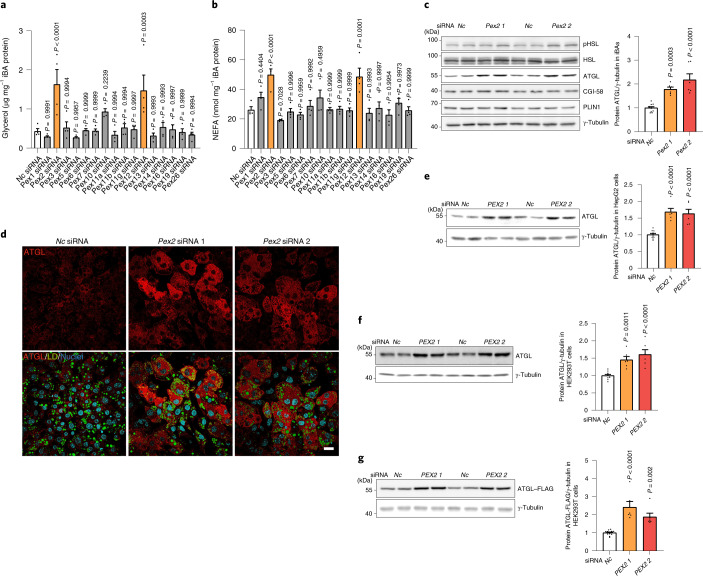
PEX2 downregulation increases iBA lipolysis and ATGL protein levels in various cell types via reduced poly-ubiquitination. **a**,**b**, Levels of glycerol and NEFAs in starvation medium released by iBAs in basal state (*n* = 4; *F* = 6.491 (**a**) and 6.884 (**b**)). **c**, IB of HSL^Ser660ph^, HSL, ATGL, CGI-58, PLIN1 and γ-tubulin in iBAs 72 h after *Pex2* knockdown (*n* = 6 in *Pex2* and 12 in *Nc* siRNA, *F* = 27.6). **d**, Representative immunofluorescence (IF) result of ATGL in iBAs 72 h after *Pex2* knockdown. ATGL in red, LDs in green and nuclei in blue. Scale bar, 20 μm. Experiments were repeated four times. **e**, IB of ATGL and γ-tubulin in HepG2 cells 48 h after *PEX2* knockdown (*n* = 6 in *Pex2* and 10 in *Nc* siRNA, *F* = 23.94). **f**,**g**, IB of endogenous ATGL or ectopically expressed ATGL–FLAG and γ-tubulin in HEK293T cells 48 h after *PEX2* knockdown (*n* = 6 in *Pex2* and 12 in *Nc* siRNA; *F* = 17.66 (**f**) and 20.9 (**g**)). Results are shown as the mean ± s.e.m. and analysed using ANOVA with Dunnett correction for multiple comparisons between control and other groups. Statistical differences are indicated by exact *P* values. Source data

**Extended Data Fig. 1:**
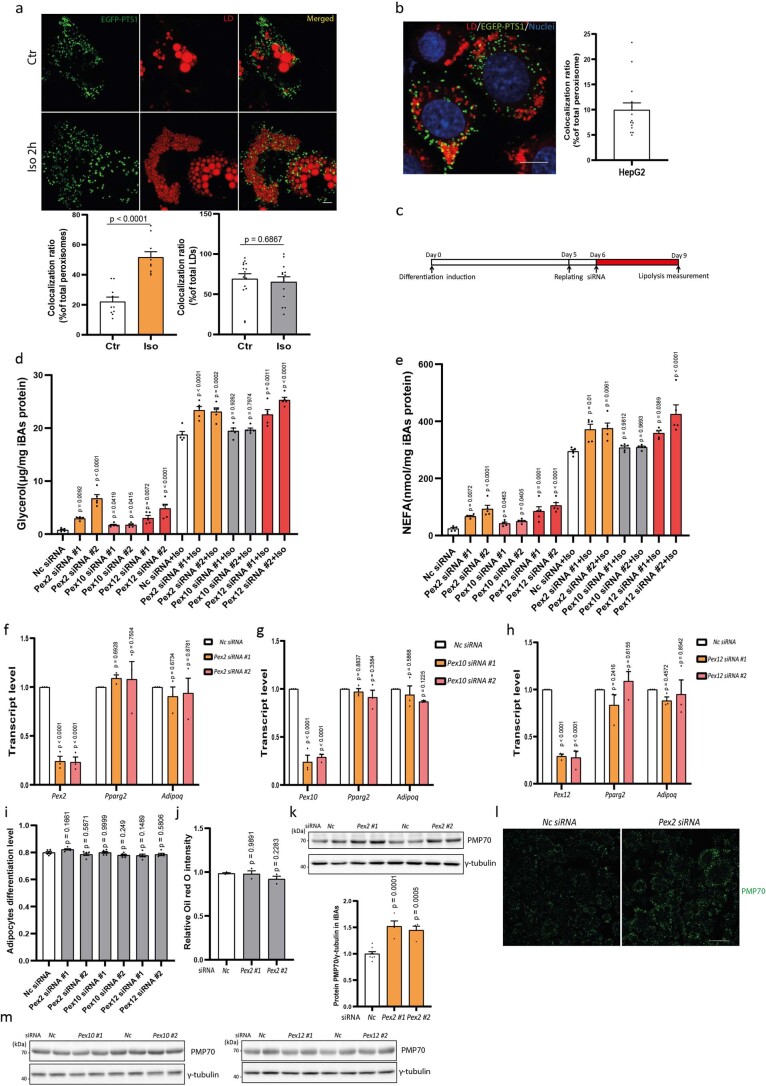
PEX2/10/12 downregulation increases iBAs lipolysis without effects on differentiation levels. (a) Quantification of peroxisomes in the proximity to LDs and LDs in proximity to peroxisomes in iBAs at basal state and stimulated state (peroxisome quantification, cell number = 10; LD quantification, cell number = 14 in control and 15 in Iso treatment). LD labelled by LipidTOX Deep Red dye (red) and peroxisomes labelled by EGFP-PTS1 (green). Scale bar, 5 μm. (b) Quantification of peroxisomes in the proximity to LDs in HepG2 cells (Cell number = 15). LD labelled by LipidTOX Deep Red dye (red) and peroxisomes labelled by EGFP-PTS1 (green). Scale bar, 10 μm. (c) Differentiation and screening strategy in iBAs. A pool of three different duplexes was utilized to knock down individual PEX target. (d-e) IBAs were transfected with siRNAs to knock down *Pex2/10/12*. After 72 h, lipolysis was determined as level of glycerol and NEFA released into starvation medium during 2 hours at basal state and 1 hour at stimulated state induced by 1 μM Iso (N = 5, F = 21.58 in basal state and 15.38 in stimulated state in d; F = 11.81 in basal state and 8.797 in stimulated state in e). (f-h) IBAs were transfected with siRNAs to knock down *Pex2/10/12*. After 72 h, *Pex2/Pex10/Pex12*, *Pparg2* and *Adipoq* transcripts were quantified by qPCR (N = 3). (i) IBAs were transfected with siRNAs to knock down *Pex2/10/12*. After 72 h, levels of adipocyte differentiation were determined via high-content imaging (N = 6, F = 4.51). (j) IBAs were transfected with siRNAs to knock down *Pex2/10/12*. After 72 h, cells were stained by Oil Red O and the latter was extracted by isopropanol for quantification (N = 3, F = 1.816). (k-l) IBAs were transfected with siRNAs to knock down *Pex2*. After 72 h, peroxisome mass was analyzed by IB or IF via peroxisomal membrane protein PMP70 (N = 4 in *Pex2* and 8 in *Nc* siRNA, F = 22.61). Scale bar, 40 μm. Repeated 3 times in l. (m) IBAs were transfected with siRNAs to knock down *Pex10/12*. After 72 h, peroxisome mass was analyzed by IB as indicated. Repeated 3 times. Results are shown as mean ± SEM and analyzed using Student’s two-sided t test (a) and an ANOVA test with Dunnett correction for multiple comparisons between control and other groups (d-k). Source data

**Extended Data Fig. 2:**
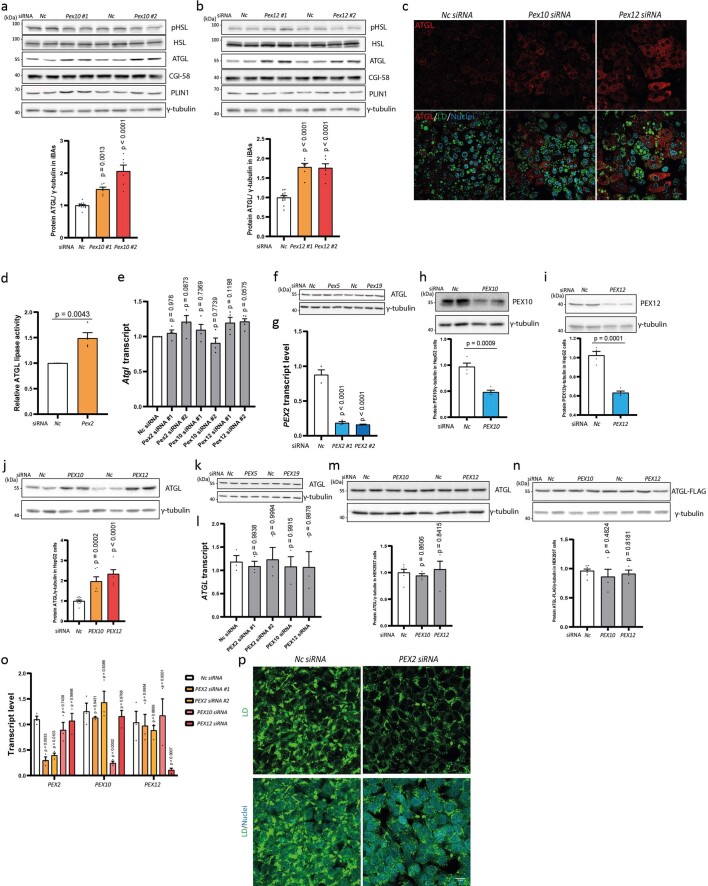
PEX2/10/12 downregulation increases ATGL protein levels independently of transcription modulation. (a-b) IB of endogenous HSL^Ser660ph^, HSL, ATGL, CGI-58, PLIN1 and γ-tubulin in iBAs 72 h after knockdown of *Pex10/12* (N = 6 in *Pex10/12* and 12 in *Nc* siRNA, F = 36.91 in a and 42.4 in b). (c) Representative IF of ATGL in iBAs 72 h after knockdown of *Pex10*/*12*. ATGL in red, LDs in green and nuclei in blue. Scale bar, 20 μm. Repeated 4 times. (d) IBAs were transfected with siRNAs to knock down *Pex2*. After 72 h, ATGL lipase activity assay was conducted using the WCE (N = 4). (e) IBAs were transfected with siRNAs to knock down *Pex2/10/12*. After 72 h, qPCR was conducted to check *Atgl* transcript (N = 4 in *Pex2/10/12* and 5 in *Nc* siRNA, F = 3.853). (f) IBAs were transfected with *Pex5* and *Pex19* siRNAs. After 72 h, IB was conducted to check protein levels by indicated antibodies. (g) Human *PEX2* knockdown efficiency was validated by qPCR 48 h after HepG2 cells were transfected with *PEX2* siRNAs (N = 3, F = 94.61). (h-i) HepG2 cells were transfected with *PEX10*/*12* siRNAs for human PEX10 and PEX12 antibodies validation by IB (N = 4). (j) IB of ATGL and γ-tubulin in HepG2 cells 48 h after knockdown of *PEX10/12* (N = 6 in *PEX10/12* and 12 in *Nc* siRNA, F = 26.32). (k) HepG2 cells were transfected with *PEX5* and *PEX19* siRNAs. After 72 h, IB was conducted to check protein levels by indicated antibodies. (l) HepG2 cells were transfected with siRNAs to knock down *PEX2/10/12*. After 48 h, qPCR was conducted to quantify *ATGL* transcript (N = 3, F = 0.1052). (m) IB of ATGL and γ-tubulin in HEK293T cells 48 h after knockdown of *PEX10/12* (N = 4 in *PEX10/12* and 6 in *Nc* siRNA, F = 0.3977). (n) IB of ectopically expressed ATGL-FLAG and γ-tubulin in HEK293T cells 48 h after knockdown of *PEX10/12* (N = 4 in *PEX10/12* and 8 in *Nc* siRNA, F = 0.6143). (o) *PEX2/10/12* knockdown efficiency was determined by qPCR 48 h after transfecting HEK293T cells by siRNAs (N = 3). (p) HEK293T cells were transfected with siRNAs to knock down *PEX2*. After 72 h, cells were fixed for LDs staining. LDs are marked in green and nuclei in blue. Scale bar, 20 μm. Repeated 4 times. Results are shown as mean ± SEM and analyzed using Student’s two-sided t test (d, h, i) and an ANOVA test with Dunnett correction for multiple comparisons between control and other groups (a, b, e, g, j-o). Source data

Peroxisomal β-oxidation accounts for a substantial part of the overall cellular FA degradation within a cell^3^ and the physical interaction between LDs and peroxisomes has been shown to facilitate LD-derived FA trafficking into peroxisomes^16^. Based on this interaction, we hypothesized that a functional crosstalk exists between both organelles to regulate the degradation of FAs to maintain lipid homeostasis. To test this hypothesis, we utilized murine immortalized brown adipocytes (iBAs) and HepG2 cells, because of their abundant content of both peroxisomes and LDs^18,19^. First, we determined peroxisome and LD distribution in both cell types and we observed, as reported previously, close contacts between the two organelles^16,20^ (Extended Data Fig. 1a,b). To investigate a possible functional crosstalk between both organelles, we studied the effect of PEX proteins, which are key mediators of peroxisomal function, on lipolysis by utilizing a small-scale screen in iBAs (Extended Data Fig. 1c). We observed that both *Pex2/12* depletion increased glycerol levels and FA release (Fig. 1a,b), suggesting an elevated lipolytic activity. A validation using different single short-interfering RNAs (siRNAs) confirmed that depletion of *Pex2/10/12* enhanced basal lipolysis, whereas *Pex2/12* knockdown enhanced stimulated lipolysis (Extended Data Fig. 1d,e). Peroxisome proliferator-activated receptor-γ 2 (*Pparg2*), adiponectin (*Adipoq*) messenger RNA (Extended Data Fig. 1f–h) as well as the overall rate of differentiation did not change in response to the depletion of *Pex2/10/12* (Extended Data Fig. 1i,j), suggesting that the observed effect is not due to alterations in the adipogenic process. Similarly, we observed a change in peroxisomal mass after PEX2 ablation, but not after PEX10/12 depletion (Extended Data Fig. 1k–m), which suggests that changes in peroxisomal mass are not required to affect the lipolytic process. To identify the mechanism by which *Pex2/10/12* depletion could induce lipolysis, we analysed several key regulators of lipolysis (Fig. 1c). Among the tested candidates only ATGL protein, a lipase reported to regulate lipolysis at both basal and activated state^21^, was significantly increased upon *Pex2/10/12* ablation (Fig. 1c and Extended Data Fig. 2a,b), which was further confirmed by immunostaining (Fig. 1d and Extended Data Fig. 2c). In accordance with this observation, ATGL activity levels were increased upon *Pex2* knockdown (Extended Data Fig. 2d). Notably, *Atgl* transcript levels remained unchanged (Extended Data Fig. 2e), suggesting that ATGL protein levels are modulated in a post-transcriptional manner. Moreover, this regulatory process is limited to PEX2/10/12, as other peroxins such as PEX5 and PEX19 did not affect ATGL levels (Extended Data Fig. 2f,k). This functional crosstalk between peroxisomes and lipolysis from LDs was confirmed in both HepG2 and HEK293T cells (Fig. 1e–g and Extended Data Fig. 2g–p), suggesting that it constitutes a conserved regulatory mechanism.

### PEX2 regulates ATGL turnover via poly-ubiquitination

**Fig. 2:**
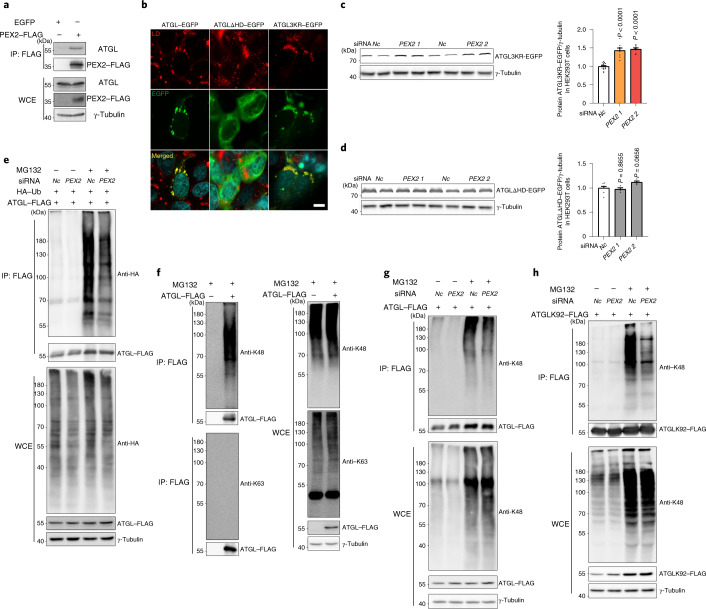
PEX2 modulates ATGL protein levels via K48-linkage poly-ubiquitination. **a**, Co-IP conducted in HEK293T whole cell extract (WCE) via FLAG antibody 48 h after expressing PEX2–FLAG. Co-IP was analysed by IB using the indicated antibodies. Experiments were repeated three times. **b**, Representative images of wild-type ATGL–EGFP, ATGLΔHD–EGFP and ATGL3KR–EGFP distribution in HEK293T cells treated with 400 μM oleic acid (OA). LDs were stained by LipidTOX Deep Red dye and nuclei were stained by 4,6-diamidino-2-phenylindole (DAPI) (blue). Scale bar, 10 μm. Experiments were repeated three times. **c**,**d**, IB of ectopically expressed ATGL3KR–EGFP and ATGLΔHD–EGFP in HEK293T cells 48 h after *PEX2* siRNA transfection (*n* = 8 in *PEX2* and 16 in *Nc* siRNA, *F* = 3.644 (**c**); *n* = 4 in *PEX2* and 8 in *Nc* siRNA, *F* = 52.67 (**d**)). **e**, HEK293T cells were co-transfected by plasmids to express ATGL–FLAG and HA–Ub, followed by siRNA transfection. After 48 h, IP and IB were conducted to detect the ubiquitination pattern. Experiments were repeated three times. **f**, HEK293T cells were transfected by the ATGL–FLAG plasmid, followed by IP to enrich ATGL–FLAG for poly-ubiquitination type analysis via K48- or K63-linkage poly-ubiquitination antibodies. Experiments were repeated three times. **g**,**h**, HEK293T cells were transfected by ATGL–FLAG (**g**) and ATGLK92–FLAG (**h**) plasmids, followed by siRNA transfection. After 48 h, IP was conducted to enrich ATGL–FLAG or ATGLK92–FLAG for K48-linkage poly-ubiquitination pattern detection. Experiments were repeated three times. Results are shown as the mean ± s.e.m. and analysed using ANOVA with Dunnett correction for multiple comparisons between control and other groups. Source data

**Extended Data Fig. 3:**
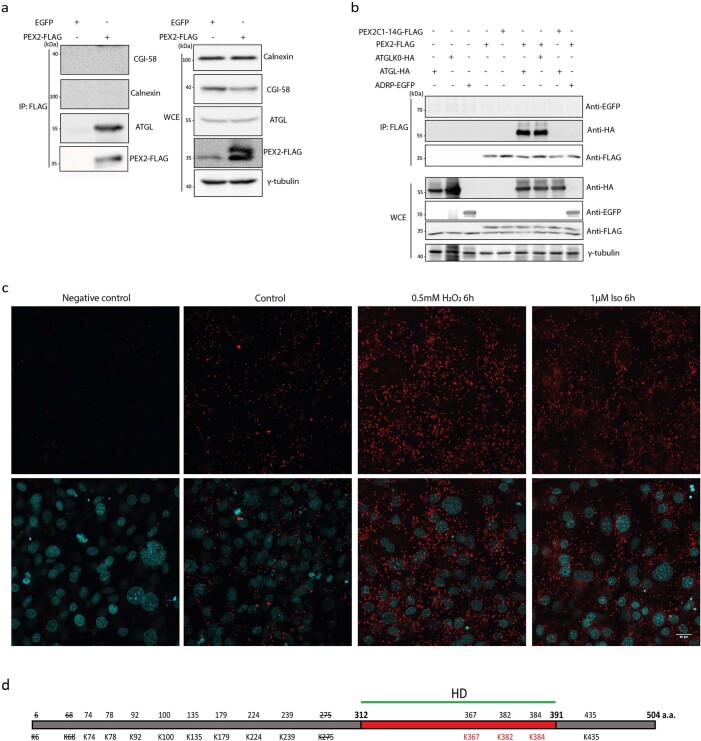
PEX2 and ATGL proteins interact physically. (a) Co-IP conducted in HEK293T WCE via FLAG antibody 48 h after expressing PEX2-FLAG. Co-IP was analyzed by IB using indicated antibodies. Repeated 3 times. (b) Co-IP conducted in HEK293T WCE via FLAG antibody 48 h after expressing indicated constructs. Co-IP was analyzed by IB using indicated antibodies. Repeated 3 times. (c) Direct interaction of ATGL and PEX2 in PEX2-FLAG-EGFP expressing iBAs was checked via proximity ligation assay (PLA) at different conditions. Upper panel represents the PEX2 and ATGL interaction. Lower panel is the nuclei (blue) merged with interaction intensity between PEX2 and ATGL. Repeated 3 times. (d) Positions of lysine residues and hydrophobic domain (HD) in human ATGL protein. Three lysine residues with strikethrough are not conserved in murine ATGL. Source data

To uncover the mechanism by which peroxisomal function is linked to the control of lipolysis through modulation of ATGL protein levels, we first analysed whether the effect was due to a direct interaction of PEX2 and ATGL. To address this point, we expressed FLAG-tagged human PEX2 (PEX2–FLAG) and induced LD formation by addition of oleic acid, followed by immunoprecipitation (IP) of PEX2. Ectopically expressed PEX2 protein levels were quite low even after transient transfection (Fig. 2a and Extended Data Fig. 3a), possibly due to a tight regulation by proteasomal degradation^10^. In spite of the low PEX2 levels, we were able to identify endogenous ATGL and ectopically expressed ATGL–FLAG, but not other LD-associated proteins such as CGI-58 and adipose differentiation-related protein (ADRP), as interaction partners of PEX2 (Fig. 2a and Extended Data Fig. 3a,b). The direct interaction between ATGL and PEX2 was further confirmed using a proximity ligation assay (PLA) (Extended Data Fig. 3c).

As ATGL mostly localizes to the LD surface in non-adipocyte cells^22^, we next investigated whether regulation takes place at the peroxisome/LD contact sites. Therefore, we ablated the ΔHD of human ATGL to express an ATGLΔHD–enhanced green fluorescent protein (EGFP) fusion protein (Extended Data Fig. 3d)^15^ that does not target the LD surface. Furthermore, we generated a mutated ATGL in which all three lysine residues in the HD were changed to arginine (ATGL3KR–EGFP) to assess whether the lysine residues in the HD are dispensable for ATGL regulation by PEX2 (Extended Data Fig. 3d). As expected, both wild-type ATGL and ATGL3KR mainly colocalized with LDs, whereas ATGLΔHD did not (Fig. 2b). Furthermore, ATGL3KR levels were elevated upon *PEX2* ablation, whereas levels of ATGLΔHD remained unaltered (Fig. 2c,d). These data demonstrate that LD localization of ATGL is required for its post-translational regulation by PEX2.

To uncover how changes in PEX2 levels led to an increase in ATGL levels and lipolysis, we focused on ubiquitination and proteasome-targeted degradation. We observed that when ATGL–FLAG was coexpressed with haemagglutinin (HA)-tagged ubiquitin (Ub), while PEX2 expression was ablated, ATGL poly-ubiquitination was reduced both in the presence or absence of MG132, suggesting that PEX2 itself is responsible for poly-ubiquitinating ATGL to target it for proteasomal degradation (Fig. 2e). Furthermore, we showed that PEX2 regulated ATGL by K48- but not by K63-linked poly-ubiquitination (Fig. 2f,g).

**Extended Data Fig. 4:**
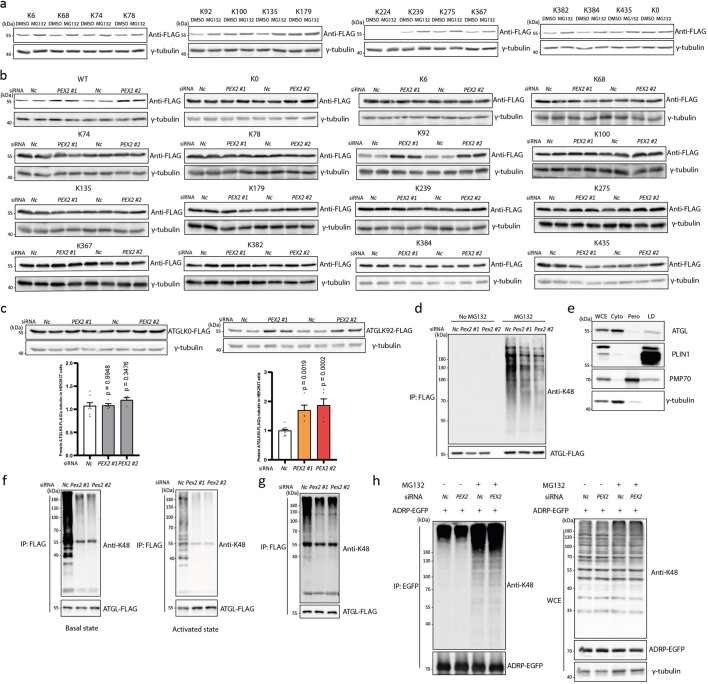
PEX2 ubiquitinates ATGL via K48-linkage poly-ubiquitination to promote its degradation in HEK293T cells. (a) ATGL lysine-only mutants were overexpressed in HEK293T cells. After 36 h, cells were treated by 10 μM MG132 for 8 h. IB was conducted to analyze protein levels after blocking proteasome dependent degradation. Repeated 2 times. (b) ATGL lysine-only mutants were overexpressed in HEK293T cells, followed by *PEX2* siRNA transfection. After 48 h, IB was conducted to determine protein levels. Repeated 2 times. (c) HEK293T cells were transfected by ATGLK0-FLAG or ATGLK92-FLAG plasmids, followed by siRNA transfection for 48 h. IB was conducted to analyze protein levels of ATGLK0-FLAG and ATGLK92-FLAG (ATGLK0-FLAG, N = 4 in *PEX2* and 8 in *Nc* siRNA, F = 0.9678; ATGLK92-FLAG, N = 5 in *PEX2* and 10 in *Nc* siRNA, F = 15.26). (d) ATGL ubiquitination was analyzed via IP and IB after stimulating ATGL-FLAG expressing iBAs with Iso. Repeated 3 times. (e) IBAs were fractionated to collect different fractions for protein analysis using indicated antibodies. Repeated 3 times. (f) ATGL-FLAG expressing iBAs were fractionated to collect LDs for IP and IB by indicated antibody. The K48-linkage poly-ubiquitination level of LD associated ATGL was analyzed from iBAs at both basal state and activated state induced by 1 μM Iso. Repeated 3 times. (g) ATGL-FLAG expressing iBAs were fractionated to collect cytosol fraction for IP and IB by indicated antibody. Repeated 3 times. (h) HEK293T cells were transfected by ADRP-EGFP plasmid, followed by *PEX2* siRNA transfection. After 48 h, IP and IB were conducted by indicated antibody to check K48-linkage poly-ubiquitination level. Repeated 3 times. Results are shown as mean ± SEM and analyzed using an ANOVA test with Dunnett correction for multiple comparisons between control and other groups. Source data

Based on our findings that PEX2 can induce K48-linked poly-ubiquitination of ATGL, we next investigated which lysine residue in ATGL is targeted by PEX2. Both human and murine ATGL contain 15 lysine residues, of which 12 are conserved (Extended Data Fig. 3d). We first mutated all lysine residues of human ATGL into arginine to generate a lysine-null mutant (K0). Subsequently, we mutated each arginine into lysine in the K0 mutant to obtain 15 site-specific lysine-only mutants. General proteasomal degradation of ATGL was dependent on multiple lysine residues (Extended Data Fig. 4a); however, only the K92-only mutant was responsive to PEX2 modulation (Fig. 2h and Extended Data Fig. 4b,c). To obtain a spatial resolution, we measured ATGL ubiquitination levels in the LD and cytosol fractions from iBAs in both a basal and stimulated state. In accordance with our previous results, we showed that PEX2 ubiquitinates ATGL in both LD and cytosol fractions to regulate its levels and lipolytic activity (Extended Data Fig. 4d–g). Instead, we observed no changes in ADRP ubiquitination levels after PEX2 depletion, suggesting specific regulation of ATGL by PEX2 (Extended Data Fig. 4h).

Taken together, our data demonstrate that PEX2 specifically poly-ubiquitinates lipolytic protein ATGL at the K92 site when ATGL distributes on the LD surface for proteasome-targeted degradation in different cell types.

### PEX2 acts as a ROS sensor in peroxisomes

**Fig. 3:**
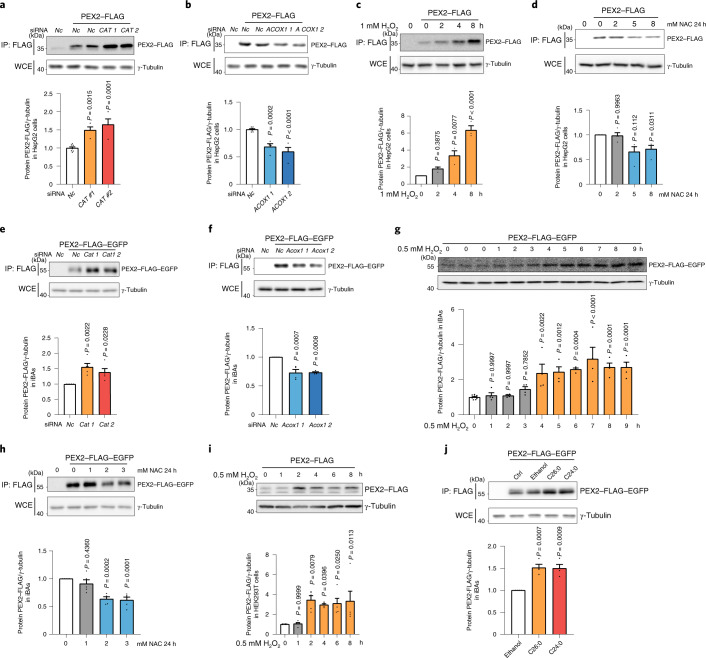
Peroxisomal β-oxidation-derived ROS regulate PEX2 protein levels. **a**,**b**, HepG2 cells with PEX2–FLAG expression were transfected with *CAT* or *ACOX1* siRNAs. After 48 h, IP and IB were conducted using the indicated antibodies (*n* = 4 in *CAT* or *ACOX1* and 8 in *Nc* siRNA, *F* = 19.79 (**a**) and 29.46 (**b**)). **c**,**d**, HepG2 cells with PEX2–FLAG expression were treated with H_2_O_2_ or NAC and collected at the indicated time points. IP and IB were conducted using the indicated antibodies (*n* = 3, *F* = 35.28 (**c**); *n* = 4, *F* = 6.717 (**d**)). **e**,**f**, iBAs with PEX2–FLAG–EGFP expression were transfected with *Cat* or *Acox1* siRNAs. After 72 h, IP and IB were conducted using the indicated antibodies (*n* = 5, *F* = 9.417 (**e**); *n* = 4, *F* = 19.9 (**f**)). **g**, iBAs with PEX2–FLAG–EGFP expression were treated with 0.5 mM H_2_O_2_ and collected at the indicated time points. IB was conducted to analyse protein levels using the indicated antibodies (*n* = 4 in H_2_O_2_ treatment and 10 in control, *F* = 10.12). **h**, iBAs with PEX2–FLAG–EGFP expression were treated with NAC at different doses for 24 h. IP and IB were conducted using the indicated antibodies (*n* = 5, *F* = 15.87). **i**, PEX2–FLAG was overexpressed in HEK293T cells by transient transfection. After 48 h, HEK293T cells were treated with 0.5 mM H_2_O_2_ and collected at the indicated time points. IB was conducted to check protein levels using the indicated antibodies (*n* = 5, *F* = 5.329). **j**, iBAs with PEX2–FLAG–EGFP expression were treated with 100 μM of hexacosanoic acid (C26:0) and lignoceric acid (C24:0) for 48 h. IP and IB were conducted using the indicated antibodies (*n* = 4, *F* = 19.54). Results are shown as the mean ± s.e.m. and analysed using ANOVA with Dunnett correction for multiple comparisons between control and other groups. Source data

**Extended Data Fig. 5:**
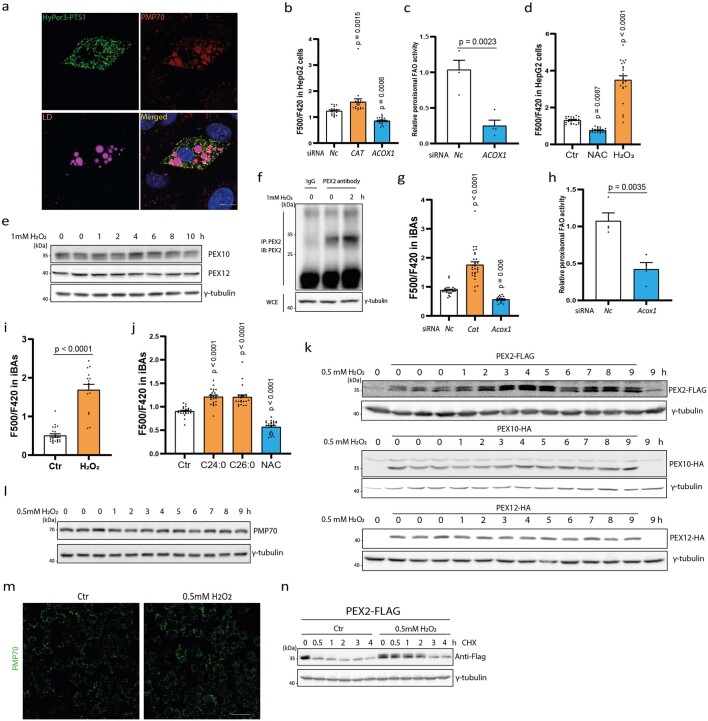
ROS regulates PEX2 protein levels. (a) Colocalization analysis of HyPer3-PTS1 with peroxisome marker (PMP70) and LDs in iBAs. Scale bar, 10 μm. Repeated 3 times. (b) Peroxisomal H_2_O_2_ levels were measured in HepG2 cells 48 h after *CAT* and *ACOX1* siRNA transfection via HyPer3-PTS1 (Cell number = 20, F = 27.56). (c) Peroxisomes were extracted from HepG2 cells after ACOX1 protein depletion to measure peroxisomal fatty acid oxidation activity (N = 4). (d) Peroxisomal H_2_O_2_ levels were measured in HepG2 cells 24 h after NAC treatment or 30 min upon H_2_O_2_ addition via HyPer3-PTS1 (Cell number = 23, F = 121.4). (e-f) HepG2 cells were treated with 1 mM H_2_O_2_ and harvested at indicated time points. IP and IB were conducted with indicated antibodies. Repeated 3 times. (g) Peroxisomal H_2_O_2_ levels were measured in iBAs cells after *Cat* and *Acox1* siRNAs transfection via HyPer3-PTS1 (Cell number = 18 in *Acox1* and 29 in *Cat&Nc* siRNA, F = 78.36). (h) Peroxisomes were extracted from iBAs after ACOX1 protein depletion to measure peroxisomal fatty acid oxidation activity (N = 4). (i-j) Peroxisomal H_2_O_2_ levels were measured in iBAs via HyPer3-PTS1 sensor after 0.5 mM H_2_O_2_, 100 μM C26:0, 100 μM C24:0 and 3 mM NAC treatment (i, cell number = 15 in H_2_O_2_ treatment and 23 in control; j, cell number = 24, F = 93). (k) HEK293T cells were transfected with plasmids to overexpress PEX2-FLAG, PEX10-HA and PEX12-HA. After 48 h, cells were treated by 0.5 mM H_2_O_2_ and harvested at indicated time points for IB via indicated antibodies. Repeated 3 times. (l-m) IBAs were treated with 0.5 mM H_2_O_2_ at indicated time points. Peroxisome mass was analyzed by IB or IF via PMP70. Scale bar, 40 μm. Repeated 3 times. (n) HEK293T cells were transfected with PEX2-FLAG plasmid. After 48 h, cells were treated by 0.5 mM H_2_O_2_ in the presence of 100 μg/ml CHX to check PEX2 degradation rate via IB. Repeated 3 times. Results are shown as mean ± SEM and analyzed using Student’s two-sided t test (c, h, i) and ANOVA test with Dunnett correction for multiple comparisons between control and other groups (b, d, g, j). Source data

PEX2 protein levels are tightly controlled and kept at a low level via a so-far-unknown mechanism^10^ (Fig. 2a and Extended Data Fig. 3a). Considering the role of peroxisomal ROS as a rheostat for peroxisomal homeostasis^23^, we hypothesized that ROS signalling might be involved in modulating PEX2 levels, possibly by stabilization of the latter. Therefore, we manipulated peroxisomal ROS levels by knocking down the peroxisomal matrix proteins CAT and ACOX1. To measure peroxisomal ROS levels, we constructed a HyPer3 protein^24^, with a PTS1 sequence (HyPer3-PTS1) for peroxisomal matrix targeting, which functions as a peroxisomal H_2_O_2_ sensor (Extended Data Fig. 5a). Using this system, we showed that ablation of *CAT* expression led to elevated peroxisomal ROS and PEX2 levels (Fig. 3a and Extended Data Fig. 5b), whereas *ACOX1* ablation reduced peroxisomal ROS and PEX2 levels (Fig. 3b and Extended Data Fig. 5b,c). To validate this association, we used H_2_O_2_ and the antioxidant *N*-acetyl-l-cysteine (NAC), which reportedly modulate global ROS levels^25^ and also affect peroxisomal ROS levels (Extended Data Fig. 5d). In line with our genetic data, we observed that H_2_O_2_ elevated PEX2 but not PEX10 and PEX12 levels (Fig. 3c and Extended Data Fig. 5e,f), whereas PEX2 protein levels were significantly reduced upon NAC treatment (Fig. 3d). These findings were confirmed in other cell types (Fig. 3e–i and Extended Data Fig. 5g–k), suggesting that modulation of PEX2 levels by peroxisomal ROS is a general regulatory concept. Although PEX2 is involved in the regulation of pexophagy, PEX2 upregulation, induced by peroxisomal ROS, did not result in a loss of peroxisome mass in iBAs (Extended Data Fig. 5l,m). Furthermore, we observed that specific targeting of peroxisomal β-oxidation by the addition of VLCFAs, which are specific substrates of this process, similarly increased peroxisomal ROS and PEX2 levels (Fig. 3j and Extended Data Fig. 5j). In addition, we showed in a chase experiment that PEX2 turnover was reduced in the presence of H_2_O_2_ (Extended Data Fig. 5n), suggesting that its stability is influenced by peroxisomal ROS levels. Taken together, these data demonstrate that PEX2 protein levels are modulated by ROS levels within the peroxisomes, which could provide a link between peroxisomal β-oxidation and ATGL-mediated lipolysis.

### PEX2 stabilization promoted by ROS-sensitive disulfide bonds

**Fig. 4:**
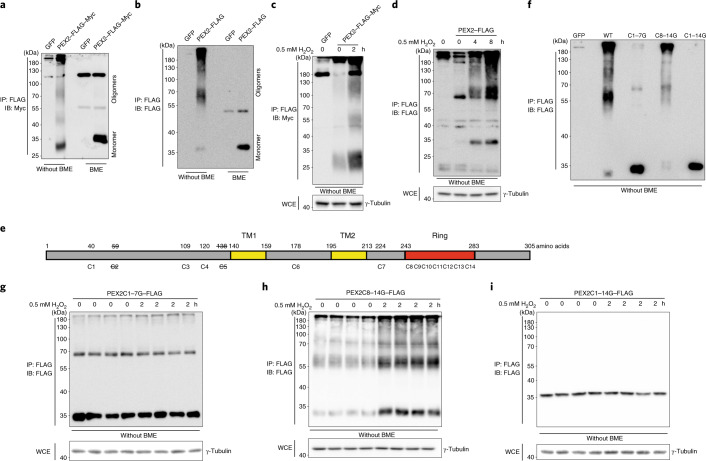
ROS regulates PEX2 protein levels via disulfide bond-mediated stabilization. **a**,**b**, HEK293T cells expressing PEX2–FLAG–Myc or iBAs expressing PEX2–FLAG were collected for IP to enrich PEX2 protein. PEX2 was eluted by Laemmli buffer with or without BME and analysed through IB via the indicated antibodies. Experiments were repeated four times. **c**,**d**, PEX2–FLAG–Myc-expressing HEK293T cells and PEX2–FLAG-expressing iBAs were treated with 0.5 mM H_2_O_2_ at different time points. IP and IB were conducted to check protein levels using the indicated antibodies at the non-reducing condition. Experiments were repeated three times. **e**, Human PEX2 scheme illustrating the position of 14 cysteines. Cysteines 1 to 7 are outside the RING domain, whereas cysteines 8 to 14 are inside the RING domain. PEX2 also contains two transmembrane motifs (TMs). Cysteines with a strikethrough do not exist in the murine PEX2. **f**, HEK293T cells were transiently transfected by plasmids to overexpress different PEX2 mutants. IP and IB were conducted at the non-reducing condition. Experiments were repeated three times. **g**–**i**, PEX2 mutants were overexpressed in HEK293T cells. After 48 h, cells were collected after 0.5 mM H_2_O_2_ treatment for 2 h. PEX2 mutants were enriched through IP for IB using the indicated antibodies. Experiments were repeated four times. Source data

**Extended Data Fig. 6:**
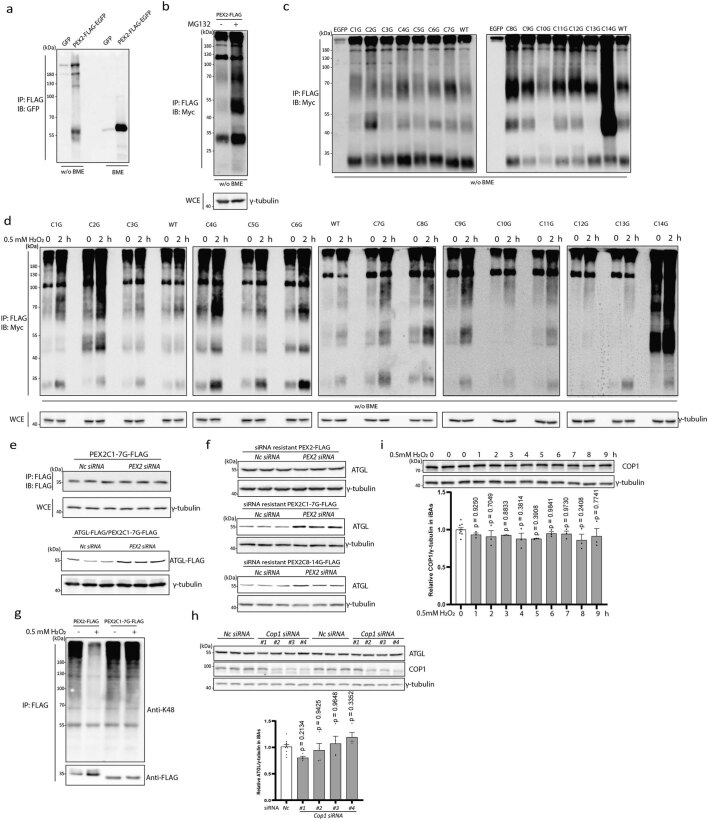
ROS promotes PEX2 stability via disulfide bonds. (a) IBAs expressing PEX2-FLAG-EGFP were collected for IP and IB by indicated antibodies. Repeated 3 times. (b) Human PEX2 was overexpressed in HEK293T cells. After treatment by 10 μM MG132 for 6 h, IP and IB were conducted to analyze PEX2 oligomerization pattern by indicated antibodies. Repeated 3 times. (c) Single cysteine to glycine mutants of human PEX2 were overexpressed in HEK293T cells. IP and IB were conducted to analyze their oligomerization patterns by indicated antibodies. Repeated 2 times. (d) Single cysteine to glycine mutants of human PEX2 were overexpressed in HEK293T cells. Cells were treated with 0.5 mM H_2_O_2_ for 2 h. IP and IB were conducted to analyze PEX2-FLAG-Myc protein by indicated antibodies. Repeated 2 times. (e-f) Wild type PEX2 or PEX2 mutants (siRNA resistant) were overexpressed in HEK293T cells or co-expressed with ATGL-FLAG, followed by siRNAs transfection. IP and IB were conducted using the indicated antibodies. Repeated 3 times. (g) PEX2 and PEX2C1-7G mutant were overexpressed in HEK293T cells, followed by 0.5 mM H_2_O_2_ treatment for 2 h. IP and IB were conducted to check K48-linkage poly-ubiquitination level. Repeated 3 times. (h-i) IBAs were transfected with *Cop1* siRNAs or treated by 0.5 mM H_2_O_2_ and harvested at the indicated time points. IB was conducted using the indicated antibodies (h, N = 4 in *Cop1* and 10 in *Nc* siRNA, F = 2.544; i, N = 3 in H_2_O_2_ treatment and 9 in control, F = 0.9765). Results are shown as mean ± SEM and analyzed using an ANOVA test with Dunnett correction for multiple comparisons between control and other groups. Source data

Disulfide bond formation is a basic mechanism to promote stabilization of various proteins. Moreover, formation of disulfide bonds can be directly regulated by ROS^26,27^. Hence, we hypothesized that ROS might promote PEX2 stability by regulating disulfide bond formation. We observed that PEX2 displayed an oligomerization pattern in the non-reducing state and alterations in retention time, which was abolished in the reducing state (Fig. 4a), demonstrating that PEX2 can form both intramolecular and intermolecular disulfide bonds in different cell lines (Fig. 4b and Extended Data Fig. 6a). When we analysed the oligomerization pattern upon H_2_O_2_ treatment, we observed that all forms of PEX2 increased (Fig. 4c,d and Extended Data Fig. 6b), suggesting that both intramolecular and intermolecular disulfide bond formation contributes to increased PEX2 stability. Human PEX2 contains 14 cysteines and 12 of them are conserved between human and murine PEX2 (Fig. 4e). By generating individual cysteine mutants, we showed that no single cysteine to glycine substitution could comprehensively disrupt the oligomerization pattern (Extended Data Fig. 6c). More notably, all mutants, albeit different in their expression levels, were stabilized by the addition of H_2_O_2_ (Extended Data Fig. 6d), suggesting that PEX2 disulfide bond formation and stabilization in response to ROS is dependent on multiple bonds. When we mutated all seven cysteines outside the RING domain into glycine (PEX2C1-7G), we observed substantially reduced levels of oligomerization, whereas the PEX2C8-14G mutant (cysteines of the RING domain) exhibited a normal oligomerization pattern (Fig. 4f). In accordance, we observed that PEX2C1-7G and PEX2C1-14G lost their responsiveness to changes in ROS levels, whereas the PEX2C8-14G protein could still be stabilized (Fig. 4g–i). In addition, we showed that cysteines outside and inside the RING domain are required for PEX2 ligase activity as ATGL upregulation induced by endogenous PEX2 depletion could not be rescued by either ectopically overexpression of PEX2C1-7G and PEXC8-14G (Extended Data Fig. 6e,f). Last, we analysed PEX2 poly-ubiquitination levels, which were reduced upon H_2_O_2_ treatment in PEX2 but not in PEX2C1-7G (Extended Data Fig. 6g). These data suggest that oligomerized PEX2 is less prone to ubiquitination or more resistant to poly-ubiquitination-mediated degradation. As COP1, an E3 ligase, has been reported to regulate ATGL in a certain cell types^28^, we analysed whether this ligase could regulate ATGL protein levels and function as a ROS sensor similar to PEX2. We observed neither increased ATGL levels upon COP1 depletion nor elevated COP1 levels in response to H_2_O_2_ exposure in iBAs, which suggests that COP1 is not required for ROS-mediated regulation of ATGL (Extended Data Fig. 6h,i). Collectively, these data demonstrate that PEX2 forms intramolecular and intermolecular disulfide bonds, which control its stability in response to alterations in ROS levels.

### Peroxisome-derived ROS regulate ATGL and lipolysis levels

**Fig. 5:**
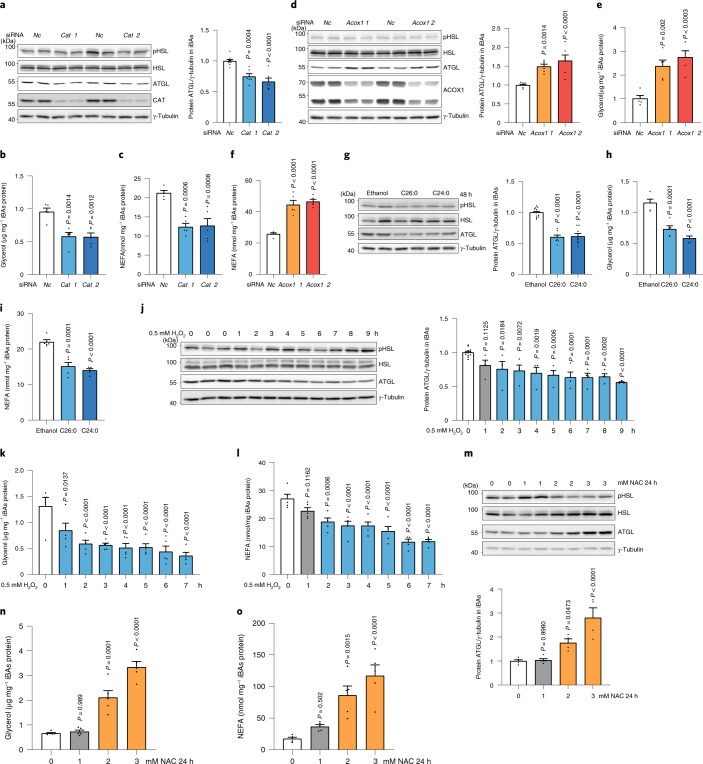
Peroxisomal β-oxidation-derived ROS regulate ATGL protein and lipolysis in iBAs. **a**–**c**, iBAs were transfected with *Cat* siRNA. After 72 h, IB, glycerol and NEFA measurement were conducted to check lipolytic proteins or lipolysis levels (*n* = 6 in *Cat* and 10 in *Nc* siRNA, *F* = 22 (**a**); *n* = 5, *F* = 13.74 (**b**) and 16.12 (**c**)). **d**–**f**, iBAs were transfected with *Acox1* siRNA. After 72 h, IB, glycerol and NEFA measurements were conducted to check lipolytic proteins or lipolysis levels (*n* = 6 in *Acox1* and 8 in *Nc* siRNA, *F* = 16.74 (**d**); *n* = 5, *F* = 16.6 (**e**) and 35.79 (**f**)). **g**–**i**, iBAs were treated with 100 μM C26:0 and C24:0 for 48 h. IB, glycerol and NEFA measurements were conducted to check lipolytic proteins or lipolysis levels (*n* = 8 in VLCFAs treatment and 13 in control, *F* = 60.22 (**g**); *n* = 5, *F* = 34.4 (**h**) and 29.5 (**i**)). **j**–**l**, iBAs were treated with 500 μM H_2_O_2_ at the indicated time points. IB, glycerol and NEFA measurements were conducted to check lipolytic proteins or lipolysis levels (*n* = 4 in H_2_O_2_ treatment and 12 in control, *F* = 7.451 (**j**); *n* = 5, *F* = 9.331 (**k**) and 15.92 (**l**)). **m**–**o**, iBAs were treated with 1 mM, 2 mM and 3 mM NAC for 24 h. IB, glycerol and NEFA measurements were conducted to check lipolytic proteins or lipolysis levels (*n* = 4 in 2 mM and 3 mM NAC treatment and 5 in 0 mM and 1 mM NAC treatment, *F* = 17.13 (**m**); *n* = 5, *F* = 48.29 (**n**) and 16.28 (**o**)). Results are shown as the mean ± s.e.m. and analysed using ANOVA with Dunnett correction for multiple comparisons between control and other groups. Source data

**Fig. 6:**
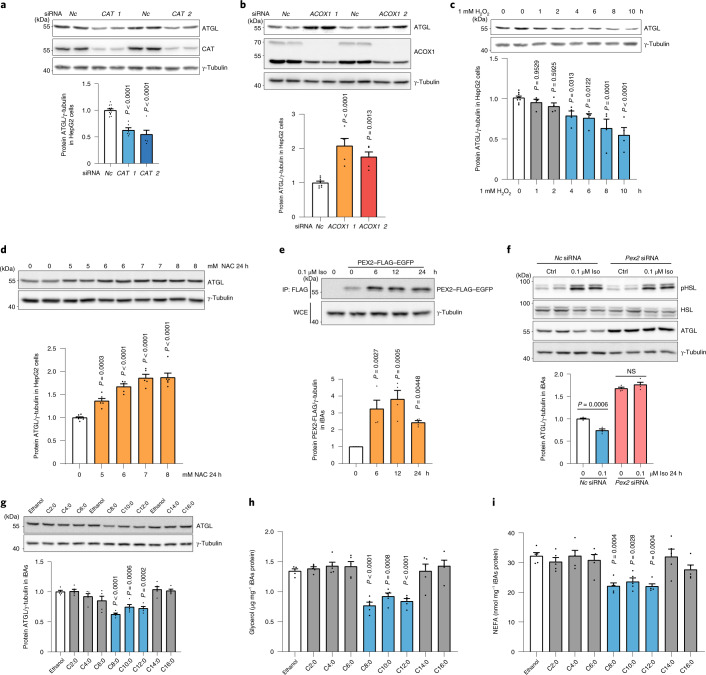
ATGL levels are regulated by ROS in HepG2 cells and repressed by overloaded FAs. **a**,**b**, HepG2 cells were transfected with *CAT* or *ACOX1* siRNA. After 48 h, IB was conducted to analyse protein levels using the indicated antibodies (*n* = 6 in *CAT* and 12 in *Nc* siRNA, *F* = 30.1 (**a**); *n* = 5 in *ACOX1* and 8 in *Nc* siRNA, *F* = 20.62 (**b**)). **c**,**d**, HepG2 cells were treated with H_2_O_2_ or NAC and collected at the indicated time points. IB was conducted to analyse protein levels using the indicated antibodies (*n* = 4 in H_2_O_2_ treatment and 9 in control, *F* = 9.689 (**c**); *n* = 5 in 6 mM and 7 mM NAC, 6 in 5 mM and 8 mM NAC and 9 in control, *F* = 47.12 (**d**)). **e**, iBAs with PEX2–FLAG–EGFP expression were treated with 0.1 μM Iso and collected at the indicated time points. IP and IB were conducted using the indicated antibodies (*n* = 4, *F* = 11.01). **f**, iBAs were transfected with *Nc* and *Pex2* siRNA. After 48 h, cells were stimulated with 0.1 μM Iso for 24 h at the indicated doses. IB was conducted to check protein levels using the indicated antibodies (*n* = 4). **g**–**i**, iBAs were treated with 100 μM of various FAs. After 48 h, cells were collected for IB analysis or lipolysis measurement was performed (*n* = 5, *F* = 14.35 (**g**), 16.51 (**h**) and 7.892 (**i**)). Results are shown as the mean ± s.e.m. and analysed using a two-sided Student’s *t*-test (**f**) or ANOVA with Dunnett correction for multiple comparisons between control and other groups (**a**–**e**,**g**–**i**). Source data

**Extended Data Fig. 7:**
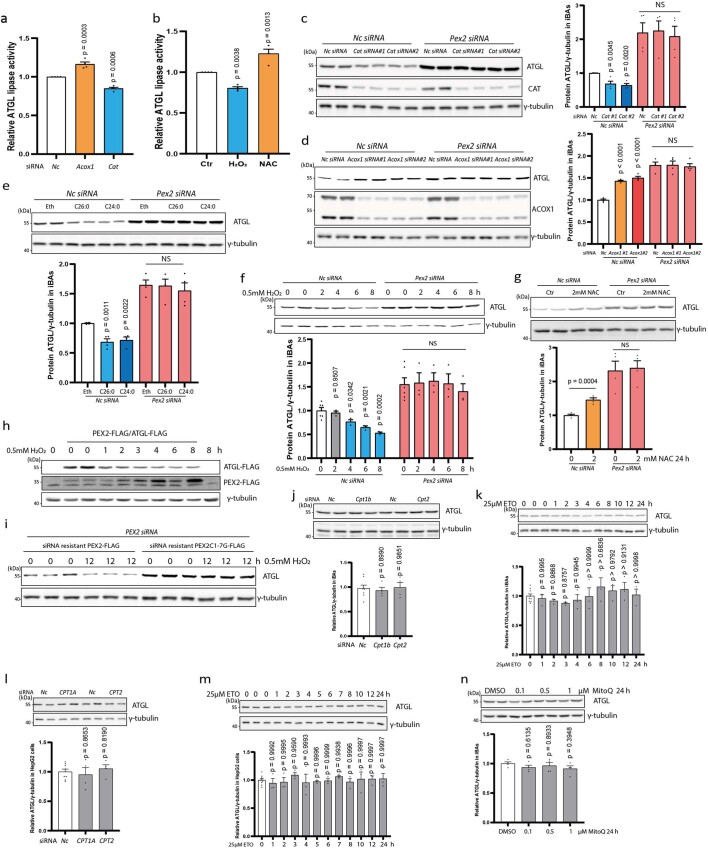
Peroxisomal β-oxidation and ROS levels regulate ATGL levels and lipolysis. (a) 72 h after knocking down *Cat* and *Acox1* in iBAs, ATGL lipase activity assays were conducted using the WCE (N = 4, F = 70.21). (b) IBAs were treated with 0.5 mM H_2_O_2_ for 9 h or 2 mM NAC for 24 h. ATGL lipase activity assays were conducted using the WCE (N = 4, F = 43.33). (c-g) IBAs were transfected with *Nc* and *Pex2* siRNAs. Under this background, cells were treated with *Cat* siRNA, *Acox1* siRNA, 100 μM C26:0 and C24:0, 0.5 mM H_2_O_2_ or 2 mM NAC, as indicated. IB was conducted to check protein levels by indicated antibodies (f, N = 3 in H_2_O_2_ treatment and 6 in control; N = 4 in c, d, e, g; F = 13.29 in *Nc* and 0.08457 in *Pex2* siRNA in c; F = 119.5 in *Nc* and 0.06723 in *Pex2* siRNA in d; F = 16.26 in *Nc* and 0.214 in *Pex2* siRNA in e). (h) PEX2-FLAG and ATGL-FLAG were co-transfected in HEK293T cells. After 48 h, cells were treated by 0.5 mM H_2_O_2_ and harvested at indicated time points. IB was conducted by indicated antibodies. Repeated 3 times. (i) Wild type PEX2 and PEX2C1-7G (siRNA resistant) were expressed in HEK293T cell under the background of endogenous PEX2 depletion. After 48 h, cells were treated by 0.5 mM H_2_O_2_ and harvested at indicated time points. IB was conducted by indicated antibodies. Repeated 3 times. (j) IBAs were transfected with *Cpt1b* and *Cpt2* siRNAs to inhibit mitochondrial fatty acid oxidation. After 72 h, IB was conducted to check protein levels by indicated antibodies (N = 4 in *Cpt1b&Cpt2* and 8 in *Nc* siRNA, F = 0.125). (k) IBAs were treated with mitochondrial fatty acid oxidation inhibitor etomoxir (ETO) at indicated time points. IB was conducted to check protein levels by indicated antibodies (N = 3 in ETO treatment and 9 in control, F = 1.016). (l) HepG2 cells were transfected with *CPT1A* and *CPT2* siRNAs to inhibit mitochondrial fatty acid oxidation. After 72 h, IB was conducted to check protein levels by indicated antibodies (N = 4 in *CPT1A&CPT2* and 8 in *Nc* siRNA, F = 0.3906). (m) HepG2 cells were treated with mitochondrial fatty acid oxidation inhibitor ETO at indicated time points. IB was conducted to check protein levels by indicated antibodies (N = 3 in ETO treatment and 9 in control, F = 0.3629). (n) IBAs were treated with mitochondria targeted antioxidant MitoQ at indicated doses for 24 h. IB was conducted to check protein levels by indicated antibodies (N = 4, F = 0.7356). Results are shown as mean ± SEM and analyzed using Student’s two-sided t test (g) and ANOVA method with Dunnett correction for multiple comparisons between control and other groups (a-f, j-n). Source data

Based on our data, we hypothesized that peroxisomal β-oxidation itself through ROS production and stabilization of PEX2 could modulate ATGL levels and thus regulate FA homeostasis. Therefore, we increased peroxisomal ROS levels by ablation of *Cat*, which resulted in a decrease in ATGL levels and activity as well as the rate of lipolysis (Fig. 5a–c and Extended Data Fig. 7a). In contrast, ATGL protein levels and activity as well as the basal lipolysis rate were elevated after *Acox1* ablation (Fig. 5d–f and Extended Data Fig. 7a). In accordance, when we supplemented cells with VLCFAs to promote exclusive peroxisomal β-oxidation, we observed a reduction in ATGL protein levels as well as basal lipolysis rates (Fig. 5g–i). Furthermore, when we manipulated peroxisomal ROS levels via H_2_O_2_ and NAC treatment, ATGL protein levels and activity and lipolysis rates were decreased by H_2_O_2_ (Fig. 5j–l and Extended Data Fig. 7b) and elevated by NAC (Fig. 5m–o and Extended Data Fig. 7b). To prove that ATGL regulation in response to changes in peroxisomal ROS levels is dependent on PEX2, we analysed ATGL regulation by *Cat* siRNA, *Acox1* siRNA, VLCFAs, H_2_O_2_ and NAC in the presence and absence of PEX2 and observed that ATGL levels were insensitive to these treatments in the absence of PEX2, demonstrating the requirement of PEX2 in this process (Extended Data Fig. 7c–g). We obtained the same results in other cell lines (Fig. 6a–d and Extended Data Fig. 7h,i), which validates the broad existence of this functional regulatory mechanisms and underscores its importance for regulating lipid homeostasis in different cell types.

As mitochondria are known to play an important role in FA oxidation, we investigated whether the interaction of lipolysis and FA oxidation is an exclusive peroxisomal feature or whether the paradigm can be extended to mitochondrial β-oxidation. Therefore, we disrupted mitochondrial FA oxidation in different cell types by CPT1 and CPT2 ablation and treated cells with the CPT1 inhibitor etomoxir to block mitochondrial FA oxidation. We observed no changes in ATGL levels after both genetic and pharmacological treatments (Extended Data Fig. 7j–m) and moreover, the mitochondria-targeted antioxidant MitoQ had no effect on ATGL levels, suggesting that mitochondrial ROS do not modulate lipolysis (Extended Data Fig. 7n). Taken together, we demonstrated that ROS derived from peroxisomal β-oxidation stabilize PEX2, which in turn leads to increased ATGL degradation and reduced lipolysis thereby regulating cellular lipid homeostasis.

### Origin of FAs as substrates for peroxisomal β-oxidation

**Extended Data Fig. 8:**
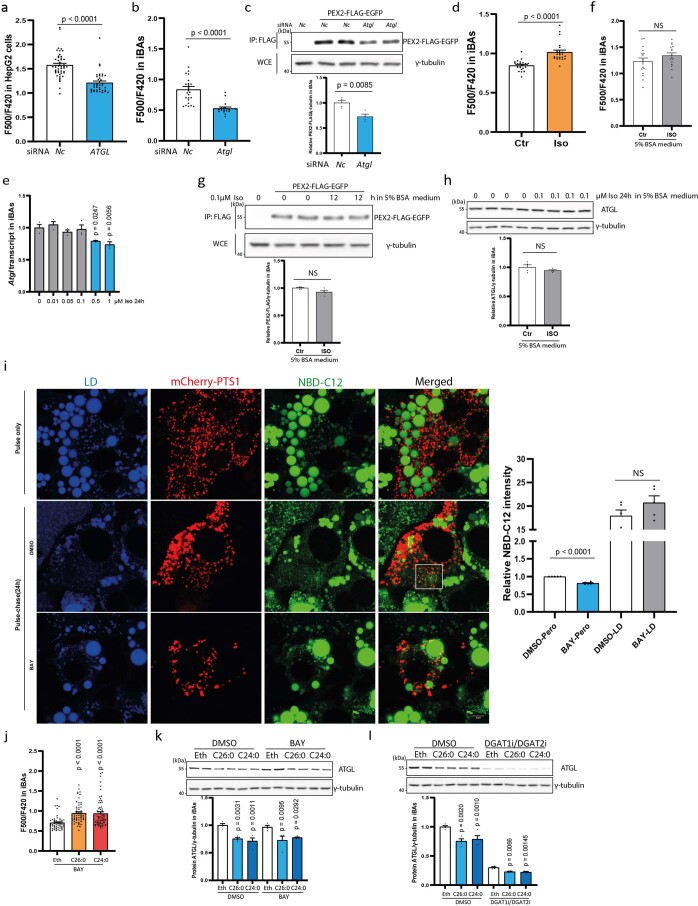
Lipolysis provides substrates for peroxisomal β-oxidation. (a-b) Peroxisomal H_2_O_2_ levels in HepG2 cells and differentiated iBAs were quantified by HyPer3-PTS1 after ATGL depletion by siRNA (a, cell number = 37 in *ATGL* and 47 in *Nc* siRNA; b, cell number = 18 in *Atgl* and 25 in *Nc* siRNA). (c) IBAs with PEX2-FLAG-EGFP expression were treated with *Atgl* siRNA and harvested 72 hours after transfection. IP and IB were conducted using the indicated antibodies (N = 4). (d) Peroxisomal H_2_O_2_ levels in differentiated iBAs were quantified by HyPer3-PTS1 6 hours after Iso treatment (cell number = 22). (e) IBAs were stimulated with Iso for 24 h at indicated doses. *Atgl* transcript was quantified by qPCR (N = 4, F = 7.57). (f) Peroxisomal H_2_O_2_ levels in differentiated iBAs were quantified by HyPer3-PTS1 6 hours after Iso treatment in the presence of 5% BSA (cell number = 16 in Iso treatment and 17 in control). (g) IBAs with PEX2-FLAG-EGFP expression were treated with 0.1 μM Iso in the presence of 5% BSA and harvested at the indicated time points. IP and IB were conducted using the indicated antibodies (N = 4). (h) IBAs were treated with 0.1 μM Iso in the presence of 5% BSA and harvested at indicated time points. IB were conducted using the indicated antibodies (N = 4). (i) Pulse-chase experiments to monitor NBD-C12 (green) trafficking from LD to peroxisome in iBAs. IBAs transfected with mCherry-PTS1 were pulsed with 10 μM NBD-C12 for 24 h, followed by lipolysis inhibitor treatment (BAY) after washing. 24 h later, cells were fixed for imaging or collected for fractionation and fluorescence measurement (N = 5). LDs were labelled by LipidTOX Deep Red dye. Scale bar, 5μm. Repeated 3 times. (j-l) IBAs were treated by 100 μM C26:0 and C24:0 for 48 h in the presence of lipolysis inhibitor (BAY) or DGAT1/2 inhibitors. Peroxisomal ROS levels were measured (cell number = 58, F = 15.71) or IB was conducted to check protein levels by indicated antibodies (N = 4; F = 15.59 in DMSO and 7.561 in BAY treatment of k; F = 16.68 in DMSO and 9.005 in DGAT1/2i treatment of l). Results are shown as mean ± SEM and analyzed using Student’s two-sided t test (a-d, f-i) or an ANOVA test with Dunnett correction for multiple comparisons between control and other groups (e, j-l). Source data

It has been reported that physical interactions between LDs and peroxisomes facilitate lipolysis-derived FA trafficking into peroxisomes^16^. Therefore, we hypothesized that the newly discovered mechanism might provide a direct feedback regulation to control FA supply via ROS-sensitive PEX2-mediated ATGL degradation. To test this hypothesis, we repressed lipolysis by ablating ATGL protein expression in different cell lines and observed a reduction of peroxisomal H_2_O_2_ levels and PEX2 protein levels (Extended Data Fig. 8a–c). In line with this, iBAs stimulated with isoproterenol (Iso) for 24 h displayed elevated peroxisomal ROS and PEX2 levels, concomitant with reduced ATGL levels (Fig. 6e,f and Extended Data Fig. 8d,e). Furthermore, the effects of Iso stimulation were attenuated after sequestering FAs released from lipolysis by the addition of bovine serum albumin (BSA)-containing medium (Extended Data Fig. 8f–h). Together, these data indicate that lipolysis-derived FAs are indeed substrates of peroxisomal β-oxidation and might thus function as a feedback regulation to modulate FA release. We further validated these findings using NBD-C12 to monitor FA trafficking from LDs to peroxisomes in a pulse-chase experiment, which demonstrated that blocking lipolysis via non-selective lipase inhibitor (BAY) reduced FA transfer into peroxisomes (Extended Data Fig. 8i).

To determine whether FAs from other pools could also regulate ATGL levels, we treated cells with lipase (BAY) or esterification (DGAT1i/DGAT2i) inhibitors in the presence of VLCFAs. We observed that VLCFA addition led to elevated peroxisomal ROS and reduced ATGL levels, even when lipolysis or esterification of FAs was blocked (Extended Data Fig. 8j–l), suggesting that under such conditions exogenous FAs can also be utilized by peroxisomal β-oxidation and in turn regulate the rate of lipolysis by modulating ATGL levels. Considering that either a lipolysis-derived FA pool or exogenous FA source is mainly composed of medium- and long-chain FAs, we further tested the effects of these FAs on ATGL and lipolysis levels. Therefore, we treated cells with FAs of different carbon lengths and observed that C8:0, C10:0 and C12:0 could reduce ATGL levels and lipolysis (Fig. 6g–i), coinciding with the substrate preference of ACOX1 (ref. ^2^). Taken together, these data demonstrate that FAs from either a lipolysis or exogenous source fuel peroxisomal β-oxidation, which in turn leads to generation of H_2_O_2_ and PEX2 stabilization, which promotes ATGL degradation, thus maintaining FA homeostasis.

### Peroxisome-derived ROS regulate hepatic ATGL levels

**Fig. 7:**
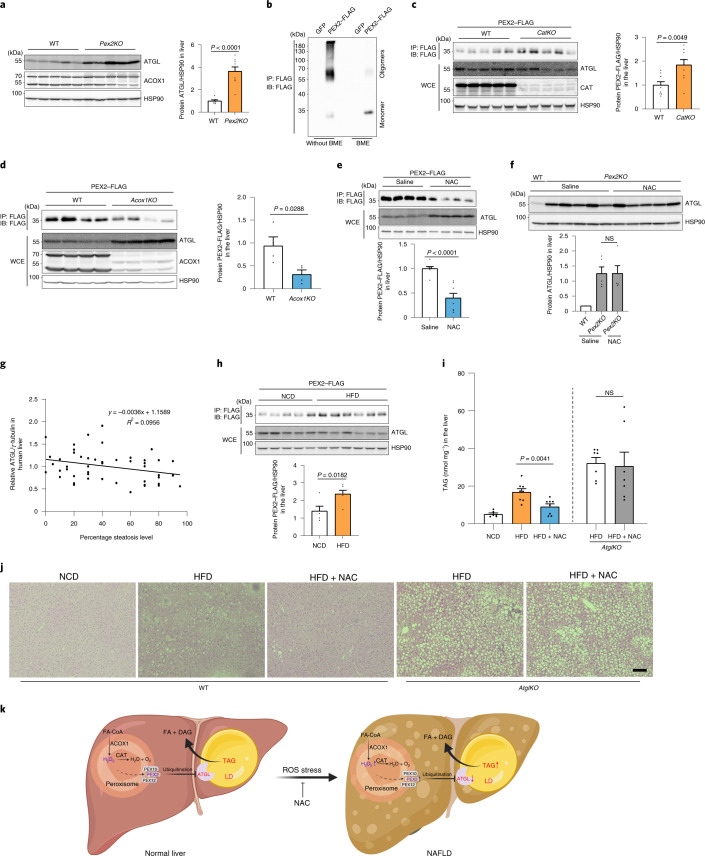
Functions of peroxisomal β-oxidation and ROS in regulating ATGL levels and TAG mobilization in the liver. **a**, Two weeks after *Pex2* knockout induction, livers were collected and hepatic proteins were analyzed by IB, as indicated (wild-type *n* = 7; *Pex2KO* *n* = 9). **b**, PEX2–FLAG was expressed for 2 weeks before liver collection and homogenization. IP and IB were conducted using the indicated antibodies. Experiments were repeated four times. **c**,**d**, PEX2–FLAG was expressed in the liver of *CatKO* or *Acox1KO* mice. IP and IB were conducted using the indicated antibodies (*n* = 9 (**c**); *n* = 4 (**d**)). **e**, PEX2–FLAG was expressed in the liver of wild-type mice. After 2 weeks, NAC was administered to the mice via intraperitoneal injection at a dose of 500 mg kg^−1^ body weight for 24 h. IP and IB were conducted using the indicated antibodies (*n* = 8). **f**, NAC was administered to the *Pex2KO* KO mice for 24 h. Livers were collected and hepatic proteins were analysed by IB (*n* = 5 for *Pex2KO* for both conditions). **g**, ATGL levels in human liver biopsies were analysed by IB and the relative ATGL levels are presented (50 human samples). *P* = 0.0372 using a Spearman test. **h**, Wild-type mice with PEX2–FLAG expression in the liver were challenged with HFD for 16 weeks. IP and IB were conducted using the indicated antibodies (*n* = 5). **i**, Wild-type and *AtglKO* mice were fed with NCD, HFD and HFD plus NAC (40 mM) in drinking water for 8 weeks. Liver lipids were extracted for TAG level determination (for wild-type mice, NCD *n* = 6, HFD *n* = 7, HFD + NAC *n* = 7; for *AtglKO* mice, *n* = 7). NCD, normal chow diet. **j**, Representative hematoxylin and eosin staining images of livers from wild-type or *AtglKO* mice fed on HFD and NAC-containing water for 8 weeks. Scale bar, 100 μm. Experiments were repeated three times. **k**, Working model illustrating the whole pathway in which peroxisomal β-oxidation generates H_2_O_2_ to stabilize PEX2 protein, resulting in increased ATGL degradation and decreased lipolysis in turn. Results are shown as the mean ± s.e.m. analysed using a two-sided Student’s *t*-test. Source data

**Extended Data Fig. 9:**
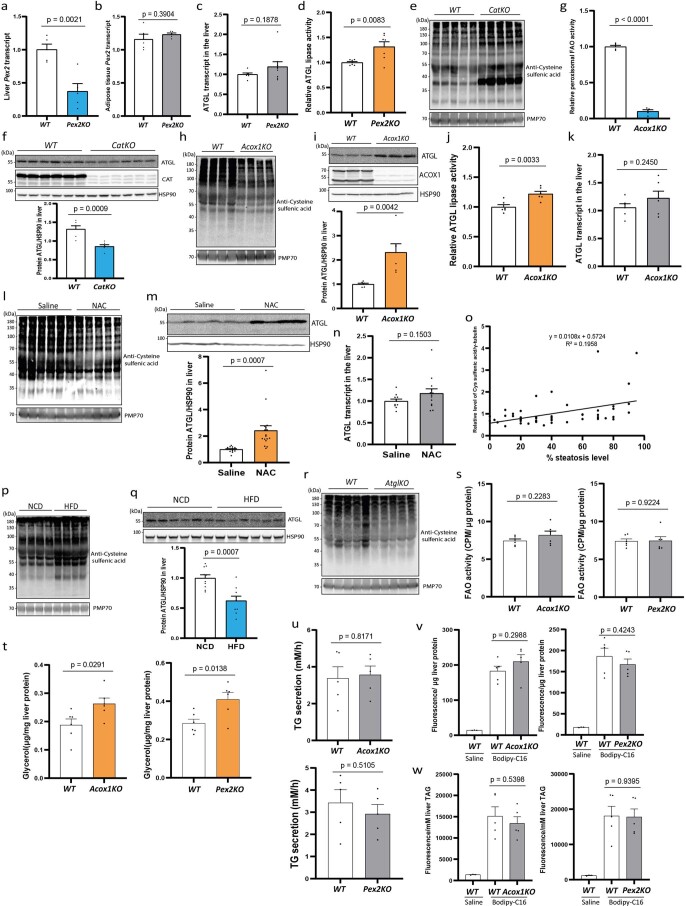
Functions of peroxisomal β-oxidation and ROS in regulating ATGL levels in liver. (a-b) *Pex2* transcript in the liver and adipose tissue of liver-specific *Pex2* knockout mice (*Pex2KO*) was quantified by qPCR (N = 5). (c) *Atgl* transcript in *Pex2* knockout liver was quantified by qPCR (*WT* N = 7; *Pex2KO* N = 9). (d) ATGL lipase activity was measured using the liver lysate from *Pex2KO* mice (N = 7). (e) Hepatic peroxisomes were extracted from the liver-specific *Cat* knockout mice to determine cysteine sulfenic acid modification levels of peroxisomal proteins. Repeated 3 times. (f) *ROSA26-LSL-spCas9* mice were injected with a pool of AAV to express *Cat* gRNA in the liver. After 2 weeks, ATGL protein levels were analyzed by indicated antibodies (N = 6). (g) *Acox*^*fl/fl*^ mice were injected with AAV-TBG-Cre virus to ablate hepatic *Acox1* (*Acox1KO*). After 2 weeks, peroxisomal β-oxidation was measured using peroxisomes from livers (N = 5). (h) Hepatic peroxisomes were extracted from the liver specific *Acox1* knockout mice to determine cysteine sulfenic acid modification levels of peroxisomal proteins. Repeated 3 times. (i) After harvesting liver from *Acox1KO* mice, hepatic ATGL levels were checked by IB with indicated antibodies (N = 6). (j) ATGL lipase activity was measured using the liver lysate from *Acox1KO* mice (N = 6). (k) *Atgl* transcript in *Acox1* knockout liver was quantified by qPCR (N = 6). (l) Peroxisomes were extracted from the livers of mice following acute NAC administration to determine cysteine sulfenic acid modification levels of peroxisomal proteins. Repeated 3 times. (m) NAC was administered acutely by intraperitoneal injection into wild type mice at dosage of 500 mg/kg BW. Hepatic ATGL levels were checked by IB with indicated antibodies (Saline N = 15; NAC N = 17). (n) Quantification of *Atgl* transcript in the livers of mice following acute NAC administration (Saline N = 11; NAC N = 13). (o) Cysteine sulfenic acid modification in human liver biopsies was analyzed by IB and relative levels of cysteine sulfenic acid modification are presented (50 human samples with different steatosis levels). P = 0.0005 by Spearman test for correlation analysis. (p) Hepatic peroxisomes were extracted from the 14 weeks HFD challenged mice to determine cysteine sulfenic acid modification levels of peroxisomal proteins. Repeated 3 times. (q) After challenging mice with HFD for 16 weeks, hepatic ATGL protein levels were analyzed by indicated antibodies (NCD N = 10; HFD N = 9). (r) *Atgl*^*fl/fl*^ mice were injected with AAV-TBG-Cre virus to knock out *Atgl* in liver. After 2 weeks, hepatic peroxisomes were extracted from these mice to determine cysteine sulfenic acid modification levels of peroxisomal proteins. Repeated 3 times. (s) Fatty acid oxidation was measured in liver homogenate from *Acox1KO* and *Pex2KO* mice using ^14^C-labelled palmitate as substrate (N = 6). (t) *Ex vivo* lipolysis was determined via the measurement of glycerol released from liver pieces dissected from *Acox1KO* and *Pex2KO* mice (N = 6). (u) Rates of TG secretion from the livers of *Acox1KO* and *Pex2KO* mice were determined by plasma triglyceride accumulation within 4 hours after tail vein injection of tyloxapol (N = 5). (v) Fatty acid uptake rate in the livers of *Acox1KO* and *Pex2KO* mice was determined via fluorescence measurement in the liver extracts 30 min after tail vein injection of Bodipy-palmitate at the dose of 10 μg/mouse (N = 5). (w) Fatty acid esterification rate in the livers of *Acox1KO* and *Pex2KO* mice was determined via fluorescence measurement in the liver lipid droplet fraction 1 h after tail vein injection of Bodipy-palmitate at the dose of 10 μg/mouse (N = 5). Results are shown as mean ± SEM and analyzed using a Student’s two-sided t test. Source data

As we demonstrated that peroxisomal ROS levels regulate ATGL protein as well as lipolysis levels in different cell types by modulating PEX2 stability, we next wanted to extend these data to the in vivo system to elucidate its physiological relevance. We focused on the liver because aberrant lipolysis has been reported to promote non-alcoholic fatty liver disease (NAFLD)^29,30^. Therefore, we utilized *LSL-spCas9* mice to knock out *Pex2* in hepatocytes (*Pex2KO*). In agreement with our previous data, ATGL protein and activity was substantially upregulated in the liver 2 weeks after *Pex2* depletion, independent of changes in the *Atgl* transcript levels (Fig. 7a and Extended Data Fig. 9a–d). Moreover, we observed that hepatic PEX2 displayed an oligomerization pattern, which was abolished after β-mercaptoethanol (BME) treatment (Fig. 7b), suggesting the existence of intermolecular and intramolecular disulfide bonds as well as the sensitivity of these bonds to peroxisomal ROS levels. To modulate specifically peroxisomal ROS levels, we generated liver-specific *Cat* and *Acox1* knockout mice. In accordance with our in vitro data, we observed that upon hepatic *Cat* ablation (*CatKO*), peroxisomal ROS levels were increased accompanied by an increase in PEX2 and a reduction in ATGL levels (Fig. 7c and Extended Data Fig. 9e,f). In contrast, in liver-specific *Acox1* knockout mice (*Acox1KO*) we observed reduced peroxisomal ROS and PEX2 levels and increased ATGL levels (Fig. 7d and Extended Data Fig. 9g–j), whereas *Atgl* transcript levels remained unchanged (Extended Data Fig. 9k).

To analyse whether the effect of peroxisomal ROS on ATGL regulation was dependent on hepatic PEX2, we used NAC to modulate ROS levels pharmacologically. We observed reduced hepatic peroxisomal ROS levels upon acute administration of NAC for 24 h (Extended Data Fig. 9l); in line with our in vitro data, we showed that this led to a reduction in hepatic PEX2 levels, which was concomitant with elevated ATGL levels (Fig. 7e and Extended Data Fig. 9m), independent of transcriptional changes (Extended Data Fig. 9n). Furthermore, NAC failed to induce ATGL in the livers of *Pex2KO* mice (Fig. 7f), demonstrating that peroxisomal ROS-dependent PEX2 stabilization is required to regulate ATGL protein levels in vivo.

### Peroxisome-derived ROS regulate hepatic steatosis

It is widely acknowledged that oxidative stress is elevated in steatotic livers and contributes to the progression of NAFLD^31^. Therefore, we next investigated whether peroxisomal ROS levels were elevated and linked to reduced ATGL levels, which might contribute to the observed lipid deposition in NAFLD. Therefore, we first analysed global ROS levels by measuring cysteine sulfenic acid modifications in human liver biopsies of two NAFLD cohorts and observed a tendency for increased ROS stress in livers with a higher grade of steatosis (Extended Data Fig. 9o). Moreover, we observed a correlation with ATGL protein levels and grade of steatosis (Fig. 7g), suggesting that this pathway is altered in NAFLD and might contribute to the progression of this disease.

**Extended Data Fig. 10:**
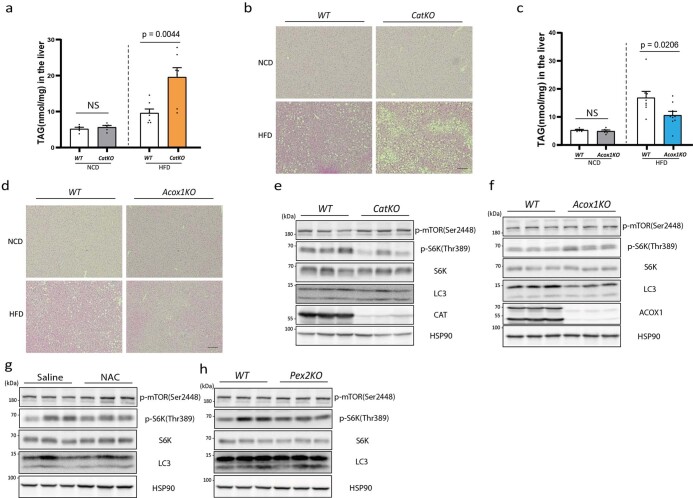
Functions of peroxisomal β-oxidation and ROS in regulating TAG mobilization in liver. (a) *ROSA26-LSL-spCas9* mice were injected with a pool of AAV to express *Cat* gRNA in the liver. After 2 weeks on NCD or 8 weeks on HFD, hepatic lipids were extracted for TAG content determination (NCD N = 5 in both genotypes; HFD N = 7 in both genotypes). (b) Representative H&E staining images of livers from liver specific *Cat* knockout mice fed on NCD or HFD for 8 weeks. Scale bar, 100μm. Repeated 3 times. (c) *Acox1*^*fl/fl*^ mice were injected with AAV-TBG-Cre virus to knock out *Acox1* in the liver. After 2 weeks on NCD or 8 weeks on HFD, hepatic lipids were extracted for TAG content determination (NCD N = 6 in both genotypes; HFD *WT* N = 8 and *Acox1KO* N = 9). (d) Representative H&E staining images of livers from liver specific *Acox1* knockout mice fed on NCD or HFD for 8 weeks. Scale bar, 100μm. Repeated 3 times. (e-h) Lipophagy/autophagy in the livers was analyzed via IB using surrogate autophagy markers, as indicated. Repeated 3 times. Results are shown as mean ± SEM and analyzed using a Student’s two-sided t test. Source data

To test the importance of this pathway for NAFLD progression, we challenged mice with a high-fat diet (HFD) to induce hepatic steatosis. In line with human data, we observed that steatotic livers displayed increased peroxisomal ROS levels as well as elevated PEX2 levels, whereas ATGL levels were reduced (Fig. 7h and Extended Data Fig. 9p–r). To test whether modulation of the identified pathway could be used to intervene in the progression of steatosis, we first analysed hepatic lipid metabolism after knocking out *Acox1* and *Pex2*. We observed upregulation of liver lipolysis, whereas global FA oxidation, TG secretion, FA uptake and esterification remained unaltered (Extended Data Fig. 9s–w). Furthermore, we induced *Cat* knockout in murine hepatocytes after 6 weeks on an obesogenic diet. This intervention led to elevated peroxisomal ROS levels and induced lipid deposition in the liver (Extended Data Fig. 10a,b). In contrast, liver-specific *Acox1* knockout mice, in accordance with previous reports^32^, exhibited a decrease in hepatic lipid deposition in response to HFD challenge (Extended Data Fig. 10c,d). To analyse whether observed changes in hepatic lipid accumulation were due to increased lipophagy, we analysed surrogate markers for lipophagy in our short-term knockout model and observed no alterations, suggesting a predominant role of lipolysis in the regulation of steatosis due to changes in peroxisomal ROS levels (Extended Data Fig. 10e–h). However, the respective contributions of lipolysis and lipophagy regulation in short-term and long-term knockout models, as well as their possible crosstalk, will require additional assessments at different time points. To demonstrate whether the protective effect of hepatic ROS reduction is dependent on lipolysis, we utilized NAC, which has been reported to alleviate HFD-induced metabolic disorders both in mice and in patients with NAFLD^33,34^, albeit through an unknown mechanism. Therefore, we challenged wild-type mice with an HFD while drinking water was supplemented with NAC. After 8 weeks of treatment, we observed a significant reduction of hepatic TAG level (Fig. 7i,j). Notably, NAC failed to alleviate hepatic TAG accumulation in the absence of ATGL (*Atgl* KO) (Fig. 7i,j), demonstrating that NAC-mediated reduction of hepatic TAG accumulation requires the presence of ATGL. These data demonstrate that ATGL upregulation in the liver due to decreased ROS levels as a result of impaired peroxisomal β-oxidation or through pharmacological intervention by NAC promotes mobilization of TAG stores, thus preventing lipid build-up and progression of steatosis (Fig. 7k).

Due to the complex nature of the identified regulatory process, our study exhibits several limitations. From published reports^10^ and our work it is evident that cellular PEX2 protein levels are strictly controlled, although this factor is crucial for peroxisome biogenesis and pexophagy. This fact limits the analysis of endogenous PEX2 levels especially in small biopsy samples. To analyse PEX2 in human steatosis, samples from hepatic surgery might be used by enriching PEX2 levels via IP. Given the prominent role of peroxisomal FA oxidation, it is interesting to note that it functions as a sensor to control cellular lipolysis in basal and activated states, whereas regulation of FA uptake and esterification, triglyceride secretion and global FA oxidation were not affected. It should be noted, however, that other metabolic pathways might be regulated through ROS-mediated PEX2 stabilization as well as the compensation of mitochondrial FA oxidation due to defective peroxisome function. As mitochondrial FA oxidation accounts for 90% of the overall FA oxidation capacity^3^, it is possible that this constitutes the explanation for why we did not observe any changes when *Acox* was ablated in vivo. If PEX2, owing to its tight regulation, is the responsible E3 ligase, it might be possible to identify other pathways by analysing the substrates of PEX2 in an unbiased manner. On the other hand, at the molecular level, ATGL protein might be regulated by other E3 ligases in addition to PEX2 to regulate FA supply to meet physiological demand.

The process of lipolysis occurs essentially in all cells and tissues in the body as it provides substrates for energy production and biosynthetic pathways. Here, we provide evidence that peroxisomal β-oxidation functions as a new sensor of FAs that regulates lipolysis via an intricate pathway to control ATGL protein levels. Few studies have hinted at such lipolysis feedback regulation by FAs dating back to 1965 (ref. ^35^). It should be noted that the study of such a mechanism is complicated by the fact that FAs via different pathways can modulate not only gene expression^36,37^, but also control insulin signalling, which in turn would affect lipolysis by modulating HSL activity^38^. Considering the fact that lipotoxicity is an important factor in the development of metabolic diseases due to uncontrolled lipolysis, the identified crosstalk between peroxisomes and LDs, which intricately maintains FA levels to meet cellular energy demands, might be utilized to treat not only NAFLD but also other metabolic disorders.

## Methods

### Cell culture and transfection

iBAs were used as described previously^39^. Once preadipocytes reached confluence, adipogenesis was induced by differentiation medium containing 3-isobutyl-1-methylxanthine (500 μM), dexamethasone (1 μM), insulin (20 nM), T3 (1 nM) and indomethacin (125 μM) for 2 d. Afterwards, iBAs were kept in maintenance medium containing insulin (20 nM) and T3 (1 nM). At day 5, differentiated iBAs were replated. All experiments were routinely performed at day 9. For compound treatment in iBAs, Iso (Sigma-Aldrich, catalogue no. I5627), H_2_O_2_ (Sigma-Aldrich, catalogue no. 216763) and NAC (Sigma-Aldrich, catalogue no. A7250) were added to the medium at the indicated doses. C2:0 (Sigma-Aldrich, catalogue no. 695092), C4:0 (Sigma-Aldrich, catalogue no. B103500), C6:0 (Sigma-Aldrich, catalogue no. 21530), C8:0 (Sigma-Aldrich, catalogue no. O3907), C10:0 (Sigma-Aldrich, catalogue no. W236403), C12:0 (Sigma-Aldrich, catalogue no. W261408), C14:0 (Sigma-Aldrich, catalogue no. M3128), C16:0 (Sigma-Aldrich, catalogue no. P0500), C24:0 (Sigma-Aldrich, catalogue no. L6641) and C26:0 (Sigma-Aldrich, catalogue no. H0388) were all dissolved in ethanol by heating and were added to the medium at a 100 μM final concentration 48 h before collecting the cells. For the knockdown experiments, lipofectamine RNAiMAX-mediated siRNA (100 nM) transfection was performed to knock down target genes on day 6.

HEK293T cells (Abcam, catalogue no. ab255449) were cultured in high-glucose DMEM with 10% FBS and 1% penicillin/streptomycin. To overexpress human PEX2/10/12 or ATGL transiently, lipofectamine 2000 was used to deliver plasmids at 60–80% cell confluence. Cells were treated with 400 μM OA (Sigma-Aldrich, catalogue no. O1008) to induce LD formation for 24 h. For the knockdown experiments, siRNAs (100 nM) were transfected using lipofectamine RNAiMAX reagent for 24 h before LD induction by 400 μM OA-containing medium. After 24 h of LD induction, cells were collected to analyse endogenous ATGL levels. To knock down *PEX2* in the condition of ATGL–FLAG overexpression, HEK293T cells were first transfected by plasmids for 12 h, followed by siRNA transfection for 24 h and LD induction by 400 μM OA-containing medium for 24 h before collecting the cells. To check ATGL localization in HEK293T cells, plasmids were delivered into cells and cells were exposed to 400 μM OA-containing medium. One day later, cells were fixed in 4% PFA for 20 min and stained with HCS LipidTOX Deep Red Neutral Lipid Stain (Thermo Fisher, catalogue no. H34477) and Hoechst 33342 for LDs and nuclei labelling, respectively.

HepG2 cells (ATCC, HB-8065) were cultured in RPMI medium with 10% FBS and 1% penicillin/streptomycin on collagen-coated plates. HepG2 cells were induced by 400 μM OA to induce LD formation. NAC and H_2_O_2_ were added to the medium at the indicated doses and time points before cell collection. For the knockdown experiments, lipofectamine RNAiMAX reagent was used for the transfection of siRNAs (100 nM). All cell lines used in this work were regularly tested for *Mycoplasma* contamination. All siRNAs used are listed in Supplementary Table 1.

### Lipolysis measurement

For lipolysis measurement, day 9 iBAs were washed with pre-warmed PBS and then starved in phenol red-free DMEM medium (low glucose) with 1% FA-free BSA for 2 h. After medium collection, cells were treated with 1 μM Iso-containing medium. Glycerol and non-esterified FA release were measured using Free Glycerol Reagent (Sigma-Aldrich, catalogue no. F6428) and non-esterified fatty acid (NEFA) assay kit (Wako NEFA kit). Glycerol and NEFA levels were normalized to protein content.

To measure liver lipolysis, mice were starved for 6 h and dissected. Livers were cut into pieces (approximately 5 mg per piece) and washed three times with PBS. Ten liver pieces from one mouse were incubated in 0.5 ml 0.5% BSA containing RPMI medium (1 g l^−1^ glucose) for 30 min. Afterwards, liver pieces were incubated with 0.5 ml 0.5% BSA containing RPMI medium for 30 min. The medium was collected for glycerol measurements and liver pieces were homogenized in RIPA buffer for protein quantification.

### High-content imaging of adipocytes

Adipocytes cultured in 96-well opaque black plates were fixed with 4% PFA for 20 min, washed three times with PBS and stained with Bodipy (Invitrogen) for LDs and Hoechst 33432 (Cell Signaling) for nuclei. Images were captured via an automated system (Operetta; PerkinElmer) and analysed using the Harmony software by calculating the ratio of cells with lipid droplet to the total cell number as described before^39^.

### RNA extraction and quantitative PCR

Total RNA was extracted from liver samples or in vitro cultured cells using TRIzol reagent (Invitrogen) according to the manufacturer’s instructions. Genomic DNA was removed from the crude RNA samples by DNase treatment (New England Biolabs). For individual samples, 1ug of total RNA was converted into complementary DNA by using the High-Capacity cDNA Reverse Transcription Kit (Applied Biosystems, catalogue no. 4368813). Quantitative PCR was performed using the Fast SYBR Green Master Mix (Applied Biosystems, catalogue no. 4385618) on ViiA7 (Applied Biosystems) and the relative transcript levels were normalized to *36B4* expression via the ΔΔCt method. Primer sequences are listed in Supplementary Table 2.

### Virus packaging and stable cell line construction

For lentivirus packaging, the pLenti-HyPer3-PTS1, pLenti-PEX2–FLAG–EGFP or pLenti-PEX2–FLAG plasmids were transfected into the 293 LTV cell line (Cell Biolabs) together with pMD2.G (Addgene, plasmid no. 12259) and psPAX2 (Addgene, plasmid no. 12260) by polyethylenimine in OptiMEM medium. The virus-containing medium was collected 2 d later and concentrated in PEG-it Virus Precipitation Solution (SBI, catalogue no. LV825A-1) according to the manufacturer’s instructions. The concentrated lentiviruses, together with hexadimethrine bromide/polybrene (Sigma-Aldrich, catalogue no. H9268), were added to the medium to infect iBA preadipocytes or HepG2 cells, which were selected by 10 μg ml^−1^ Blasticidin (Thermo Fisher, catalogue no. A1113903).

For adeno-associated virus (AAV) packaging, we transfected AAV–TBG–Cre, AAV–TBG–green fluorescent protein (GFP), AAV-U6-NS guide RNA (gRNA)–TBG–Cre, AAV–U6–Pex2 gRNA–TBG–Cre, AAV–U6–Cat gRNA–TBG–Cre or AAV–TBG–PEX2–FLAG plasmids into 293 AAV cells (Cell Biolabs) together with AAV8 serotype helper plasmid (PlasmidFactory, pDP8.ape) using polyethylenimine in OptiMEM medium. The medium was collected 4–5 d later for concentration using the AAVanced Concentration Reagent (SBI, AAV110A-1) according to the manufacturer’s instructions. The titre of concentrated viruses was determined by quantitative PCR based on the titre determination protocol from Addgene.

### Molecular cloning

HyPer3 was cloned from the pAAV-HyPer3 vector (Addgene, plasmid no. 119183) with a PTS1 sequence in the carboxy terminus and inserted into a pLenti-CMV-MCS-BSD vector. Human ATGL–FLAG (catalogue no. RC205708) and PEX2–FLAG–Myc (catalogue no. RC218196) expression plasmids were obtained from OriGene. To build the lentivirus constructs, human PEX2 was cloned from PEX2 (catalogue no. RC218196) with a FLAG tag at the C terminus and inserted into a pLenti-CMV-MCS-BSD vector to obtain the pLenti–PEX2–FLAG construct. Murine PEX2 was cloned from an iBA cDNA library and inserted into a pEGFP–N1–FLAG vector (Addgene, plasmid no. 60360). Then PEX2–EGFP coding sequence (CDS) or PEX2 CDS alone were cloned and inserted into a pLenti-CMV-MCS-BSD vector to obtain pLenti–PEX2–FLAG–EGFP and pLenti–PEX2–FLAG constructs. To build an AAV vector for hepatic expression of murine PEX2–FLAG, murine PEX2 was cloned from a pEGFP–N1–FLAG vector with a FLAG tag at the C terminus and inserted into pAAV.TBG.PI.eGFP.WPRE.bGH (Addgene, plasmid no. 105535). For human ATGL lysine-only mutants, we first obtained the ATGL lysine-null nucleotide sequence from GenScript cloned into a pEGFP–N1–FLAG vector with a FLAG tag and stop codon at the C terminus. The Agilent QuikChange II Site-Directed Mutagenesis Kit was used to mutate single arginine into lysine residues based on the lysine-null ATGL construct. Human ATGL was cloned from ATGL–FLAG (catalogue no. RC205708) and inserted into pEGFP–N1–FLAG to obtain the ATGL–EGFP fusion protein. Three lysine residues in the homeodomain (HD) were mutated to arginine stepwise to obtain ATGL3KR–EGFP. To build a hydrophobic domain-deficient ATGL, nucleotide sequences between 1 to 936 and 1,174 to 1,512 were cloned and fused via overlapping PCR and the fragment was inserted in pEGFP–N1–FLAG to generate ATGLΔHD–EGFP. For human PEX2 cysteine mutants, individual cysteine residues were mutated to glycine using PEX2–FLAG–Myc (catalogue no. RC218196). The human PEX2 cysteine-null mutant was synthesized and cloned into a pEGFP–N1–FLAG vector with a FLAG tag and stop codon at the C terminus. By overlapping PCR, the C terminus from the wild-type PEX2 and the amino terminus from the cysteine-null PEX2 were integrated and inserted into a pEGFP–N1–FLAG with a FLAG tag and stop codon at the C terminus to generate human PEX2C1–7G–FLAG. PEX2C8–14G–FLAG was constructed likewise. ADRP–EGFP was purchased from Addgene (plasmid no. 87161). All plasmids for the cell culture work were extracted using NucleoBond Xtra-Midi Kit (Macherey-Nagel, product no. 740410.100).

### Immunoblotting, immunoprecipitation, co-immunoprecipitation and immunofluorescence

Liver samples, iBAs, HepG2 cells and HEK293T cells were homogenized in RIPA buffer (50 mM Tris-HCl, pH 7.4, 150 mM NaCl, 2 mM EDTA, 1.0% Triton X-100 and 0.5% sodium deoxycholate) with protease inhibitors (Roche) and phosphatase inhibitors (Thermo Fisher). The homogenates were centrifuged at 12,000*g* for 10 min at 4 °C to remove debris and to collect the whole cell extract. After boiling the sample with Laemmli buffer at 95 °C for 5 min, equal amounts of proteins were loaded and separated on a 12% SDS–PAGE. The proteins were transferred to nitrocellulose membrane or PVDF membrane (Bio-Rad) and incubated in ATGL (1:1,000 dilution, Cell Signaling), CGI-58 (1:1,000 dilution, Proteintech), phospho-HSL (Ser660) (1:1,000 dilution, Cell Signaling), PLIN1 (1:1,000 dilution, Cell Signaling), HSL (1:1,000 dilution, Cell Signaling), γ-tubulin (1:10,000 dilution, Sigma-Aldrich), FLAG (1:10,000 dilution, Sigma-Aldrich ; 1:1,000 dilution, Cell Signaling), PEX10 (1:1,000 dilution, Sigma-Aldrich), PEX12 (1:1,000 dilution, Abcam), CAT (1:1,000 dilution, Cell Signaling), ACOX1 (1:1,000 dilution, Abcam), phospho-mTOR (Ser2448) (1:1,000 dilution, Cell Signaling), phospho-S6K (Thr389) (1:1,000 dilution, Cell Signaling), S6K (1:1,000 dilution, Cell Signaling), LC3 (1:1,000 dilution, Cell Signaling), Calnexin (1:1,000 dilution, Cell Signaling), PEX2 (1:1,000 dilution, Thermo), EGFP (1:1,000 dilution, Abcam), COP1 (1:1,000 dilution, Abcam), PMP70 (1:10,000 dilution, Sigma-Aldrich) and HSP90 (1:1,000 dilution, Cell Signaling) antibodies. The primary antibody signal was visualized by HRP-conjugated secondary antibodies (1:10,000, Cell Signaling) and the ImageQuant system (GE Healthcare Life Sciences).

To immunoprecipitate FLAG-tagged ATGL or PEX2, liver samples, iBAs, HepG2 cells and HEK293T cells were homogenized in RIPA buffer (50 mM Tris-HCl, pH 7.4, 150 mM NaCl, 2 mM EDTA and 1.0% Triton X-100) with protease inhibitors (Roche) and phosphatase inhibitors (Thermo Fisher). After three washing steps with cold RIPA buffer, 20 μl of anti-FLAG beads (Sigma-Aldrich, catalogue no. A2220) were added and the mixture was incubated overnight at 4 °C on a rotator. Beads were washed with cold RIPA buffer six times. The proteins were eluted by boiling at 95 °C for 5 min in 2× Laemmli buffer. For the PEX2 polymerization analyses, we used a BME-free Laemmli buffer. The eluted proteins were analysed by immunoblot (IB) with K48- or K63-linkage-specific ubiquitin antibodies (1:1,000 dilution, Cell Signaling), HA (1:1,000 dilution, Cell Signaling), Myc (1:1,000 dilution, Sigma-Aldrich), FLAG (1:1,000 dilution, Cell Signaling) or ATGL (1:1,000 dilution, Cell Signaling) antibodies.

To immunostain ATGL or PMP70 in iBAs, cells were fixed with 4% PFA for 20 min and permeabilized with 0.25% PBST (Triton X100 in PBS) for 20 min. After three washes with cold PBS, cells were blocked with 1% BSA for 1 h and incubated with an ATGL antibody (1:100 dilution, Cell Signaling) or PMP70 antibody (1:100 dilution, Sigma-Aldrich) overnight at 4 °C. After three washes with cold PBS, cells were incubated with an Alexa Fluor 488- or 568-conjugated secondary antibody (1:200 dilution, Thermo Fisher) for 1 h at room temperature. Finally, cells were stained with Hoechst 33342 or LipidTOX Deep Red dye to label nuclei and LDs, respectively, after being washed three times with PBS to remove residual secondary antibody. Images were obtained using an Olympus FLUOVIEW 3000 confocal microscope and processed with ImageJ v.1.53e.

### H_2_O_2_ measurements

IBAs and HepG2 cells were infected by HyPer3-PTS1 lentivirus and live cells were imaged with an Olympus FLUOVIEW 3000 confocal microscope. For the reduced form of HyPer3-PTS1, we set excitation at 405 nm and emission between 510 and 540 nm (F420). For the oxidized form of HyPer3-PTS1, we set excitation at 488 nm and emission between 510 and 540 nm (F500)^24^. The intensity of F420 and F500 was analysed by ImageJ and the F500/F420 ratio indicated the H_2_O_2_ levels.

To analyse peroxisomal H_2_O_2_ levels in the murine liver, peroxisomes were extracted from liver pieces and suspended in RIPA buffer containing 1 mM dimedone for 30 min^40^. Non-reducing Laemmli buffer containing 100 mM maleimide was used to denature peroxisomal proteins for analysis using the sulfenic acid modified cysteine antibody (1:1,000 dilution, Sigma-Aldrich, catalogue no. ABS30). The same method was used to analyse cysteine sulfenic acid modifications of tissue lysate from human liver biopsies treated with dimedone.

### Peroxisome extraction

Peroxisomes were extracted from iBAs or HepG2 cells (3 × 10^6^ cells) following the manufacturer’s protocol (Peroxisome Isolation Kit, Sigma-Aldrich) for peroxisomal FA oxidation (FAO) and NBD-C12 pulse-chase experiments. Peroxisomes extracted from 0.5-g liver pieces were used for peroxisomal FAO and cysteine sulfenic acid modification analysis. For the FAO and NBD-C12 pulse-chase experiments, the peroxisome fraction extracted was suspended in peroxisome extraction buffer, whereas RIPA was used to lyse peroxisomal proteins for cysteine sulfenic acid modification analysis.

### FAO measurement

To measure peroxisomal FAO, peroxisomes were extracted from iBAs, HepG2 cells or livers and suspended in peroxisome extraction buffer (5 mM MOPS, pH 7.65, 250 mM sucrose, 1 mM EDTA and 0.1% ethanol). The FAO rate was measured spectrophotometrically^41^. The reaction was monitored using a Synergy Gen5 plate reader in a 200-μl system (188 μl of 50 mM Tris-HCl, pH 8.0, 2 μl 20 mM β-nicotinamide adenine dinucleotide hydrate, 0.6 μl of 0.33 M dithiothreitol, 1 μl of 1.5% BSA, 1 μl of 2% Triton X-100, 2 μl of 10 mM CoA, 2 μl of 1 mM flavin adenine dinucleotide disodium salt hydrate, 2 μl of 100 mM KCN, 0.4 μl of 5 mM C12-CoA and 1 μl of peroxisome extract).

To measure global FAO, livers were homogenized in peroxisome extraction buffer. Then, 30 μl of liver homogenate were added to the 370-μl reaction mixture (100 mM sucrose, 10 mM Tris-HCl, 5 mM KH_2_PO_4_, 0.2 mM EDTA, 80 mM KCl, 1 mM MgCl_2_, 2 mM l-carnitine, 0.1 mM malate, 0.05 mM CoA, 2 mM ATP, 1 mM dithiothreitol, 7% BSA, 5 mM palmitate and 0.4 μCi ^14^C-palmitate) in 1.5-ml tubes together with an NaOH-containing filter paper disk after 10-min centrifugation at 400*g*. After 30 min at 37 °C, the filter paper disk was transferred into a scintillation vial containing 5 ml of scintillation fluid for overnight incubation. The average counts per minute were measured using a standard scintillation counter.

### ATGL lipase activity assay

We used a simplified ATGL activity assay to measure ATGL activity in iBAs and liver as described, with some modifications^42^. Briefly, iBAs or liver pieces were homogenized in assay buffer (50 mM Tris-HCl, pH 7.4, 150 mM NaCl and 0.1% Triton X-100), followed by sonication for 1 min. The extract was collected after centrifugation at 12,000*g* for 10 min. The assay comprised a 95-μl extract and 5 μl of 2 μM EnzChek Lipase Substrate (Life Technologies) and was performed in 96-well opaque black plates. The kinetic liberation rate of the fluorescent product (excitation 485 nm and emission 510 nm) was monitored using a Synergy Gen5 plate reader. ATGL activity was normalized to protein levels and presented as the relative value to the control.

### NBD-C12 pulse-chase assay

iBAs were treated with 10 mM NBD-C12 to incorporate the latter into the LDs^16^. After 24 h, iBAs were washed with PBS to remove residual NBD-C12 and further cultured in NBD-C12-free medium for 24 h with or without the non-selective lipase inhibitor BAY (CAY10499, Cayman Chemical). We checked NBD-C12 distribution in peroxisomes and LDs via imaging or quantification of fluorescence in extracted peroxisomes. Images were obtained using an Olympus FLUOVIEW 3000 confocal microscope and processed by ImageJ. After suspending the extracted peroxisomes into peroxisome extraction buffer, fluorescence (excitation 466 nm and emission 530 nm) was monitored using a Synergy Gen5 plate reader and presented as the relative values to controls after normalization to protein levels.

### Proximity ligation assay

PEX2–FLAG-expressing iBAs were treated differently, followed by fixation via PFA for 20 min at room temperature. A proximity ligation assay was then performed using the kit from Sigma-Aldrich (catalogue no. DUO92101), following standard protocols with mouse antibody (FLAG, F3165 from Sigma-Aldrich) and rabbit antibody (ATGL, 2138 from Cell Signaling) incubation or incubation without antibodies as the negative control. Images were obtained using an Olympus FLUOVIEW 3000 confocal microscope and processed with ImageJ.

### Patient liver biopsies

Human liver biopsies were collected in two cohorts and were analysed for data integration. The liver biopsies of one cohort were obtained during the work-up of liver disease diagnostics in the outpatient clinic of the Division of Gastroenterology and Hepatology, University Hospital Basel, Switzerland. The study was carried out in accordance with the Code of Ethics of the World Medical Association (Declaration of Helsinki 2013, seventh revision) and was approved by the local ethics committee (EKNZ 2014-362). Written informed consent was obtained from all patients. Five to ten millimeters of liver biopsy cylinders were immediately snap-frozen by immersion in liquid nitrogen and stored in liquid nitrogen vapours until processing. The human liver biopsies of the second cohort were obtained from participants of the Biological Atlas of Severe Obesity cohort, an ongoing prospective cohort study for the longitudinal assessment of metabolic outcomes after weight loss surgery (NCT01129297), which was approved by the CHRU Lille Ethical committee and were compliant with French National Ethics Committee guidelines. The study design has been described previously in detail^43^. Clinical patient information is described in Supplementary Table 3.

### Mouse experiments

All animal experiments were approved by the veterinary office of the Canton of Zurich. The mice used in the experiments were housed 2–5 littermates per cage in ventilated cages under standard housing conditions (22 °C, 40% humidity, 12-h reversed light–dark cycle, the dark phase starting at 7:00) with ad libitum access to NCD (18% protein, 4.5% fiber, 4.5% fat and 6.3% ashes, Provimi-Kliba) and water. The health status of all mice was monitored regularly based on the Federation of European Laboratory Animal Science Associations guidelines. The 8–10-week-old female C57BL/6 and liver-specific *Pex2* overexpression or knockout mice were subjected to NAC administration (500 mg kg^−1^ body weight) via intraperitoneal injection. After 24 h, mice were killed for liver collection. To express FLAG-tagged murine PEX2 in the liver, AAV–TBG–GFP or AAV–TBG–PEX2–FLAG viruses were injected into mice via tail veins at a dose of 3 × 10^11^ genome copies per mouse. To knock out *Pex2* specifically in the liver, *ROSA26-LSL-spCas9* mice (The Jackson Laboratory strain) were injected with AAV–U6–*NS* gRNA–TBG–Cre, AAV–U6–*Pex2* gRNA–TBG–Cre or AAV–U6–*Cat* gRNA–TBG–Cre virus pools to express six *Pex2* gRNAs or *Cat* gRNAs in conjunction with CAS9 in the liver. To knock out *Acox1* specifically in the liver, we utilized *Acox1* floxed mice^19^. The *Acox1* floxed mice were injected with AAV–TBG–Cre virus. For HFD, NAC was administered to C57BL/6 mice in drinking water (40 mM) for 8 weeks. For liver-specific *Atgl* knockout mice, *Atgl* floxed mice (The Jackson Laboratory strain)^44^ were injected with AAV–TBG–Cre virus. Afterwards mice were challenged with an HFD- and NAC-containing drinking water for 8 weeks. *Acox1* or *Cat* liver-specific knockout mice were challenged by HFD for 6 weeks first and injected with virus to induce knockout and kept for 2 weeks on an HFD. Overall, all HFD experiments were performed after 8 weeks of HFD feeding, whereas experiments on NCD were carried out for 2 weeks after virus injection or 24 h after acute NAC injection. Before tissue collection, all mice were starved for 6 h.

### Liver TAG determination

The weight of liver pieces (50–100 mg) was recorded before homogenization by 1 ml of chloroform:methanol (2:1) per 50 mg tissue. Homogenates were incubated at room temperature on a rotator before spinning to remove debris at 600*g* for 5 min. Then, 200 μl of saline per 50 mg of tissue was added, followed by vortexing and centrifugation at 400*g* for 5 min. The lower phase was dried after removing the upper phase. The lipids were suspended in 2% Triton X-100 solution with sonication. The TAG levels were determined (Roche Trig/GB reagent) and normalized to the weight of the liver pieces.

### Haematoxylin and eosin staining

Livers were fixed in 4% PFA in PBS for 24 h directly after tissue collection. Liver pieces were transferred to 65% ethanol and embedded in paraffin in a tissue-processing and embedding machine, followed by sectioning at 10 μm and staining with hematoxylin and eosin.

### Liver TG secretion, FA uptake and esterification

Mice were starved for 6 h and blood was sampled before tyloxapol (80 mg ml^−1^) administration via the tail vein. After 4 h, blood was sampled again. Alterations of plasma TAG levels were measured (Roche Trig/GB reagent) to calculate the TAG secretion rate.

Mice were injected with BODIPY-palmitate (10 μg per mouse) via the tail vein. At 30 min after injection, livers were dissected for homogenization in RIPA buffer. The BODIPY fluorescence of the homogenate was measured using a Synergy Gen5 plate reader and normalized to protein levels.

For FA esterification determination, 1 h after BODIPY-palmitate injection, livers were dissected for LD extraction. The fluorescence intensity of the extract was measured and normalized to the TAG levels of the LDs to calculate the esterification rate.

### Quantification and statistical analysis

For the in vivo studies, littermates were randomly assigned to treatment groups for all experiments. Sample size was determined based on previous experiments and the animal numbers used in the experiments are indicated in the corresponding figure legends. For the cell culture experiments, at least three technical replicates were used. Results are presented as the mean ± s.e.m. A two-tailed unpaired Student’s *t*-test was applied to comparisons of two groups. Analysis of variance (ANOVA) was applied to comparisons of multiple groups using a Dunnett correction post-hoc analysis. A non-parametric Spearman test was used for the correlation analysis. All statistical analyses were performed using GraphPad Prism 7 software. Statistical differences are indicated as exact *P* values.

### Reporting Summary

Further information on research design is available in the Nature Research Reporting Summary linked to this article.

## Supplementary information

Supplementary Information Supplementary Tables 1–3

Reporting Summary

## Data Availability

All raw data are available and provided as resource data. Source data are provided with this paper.

## Source data

Source Data Fig. 1 Uncropped western blot.
Source Data Fig. 1 Statistical source data.
Source Data Fig. 2 Uncropped western blot.
Source Data Fig. 2 Statistical source data.
Source Data Fig. 3 Uncropped western blot.
Source Data Fig. 3 Statistical source data.
Source Data Fig. 4 Uncropped western blot.
Source Data Fig. 5 Uncropped western blot.
Source Data Fig. 5 Statistical source data.
Source Data Fig. 6 Uncropped western blot.
Source Data Fig. 6 Statistical source data.
Source Data Fig. 7 Uncropped western blot.
Source Data Fig. 7 Statistical source data.
Source Data Extended Data Fig. 1 Uncropped western blot.
Source Data Extended Data Fig. 1 Statistical source data.
Source Data Extended Data Fig. 2 Uncropped western blot and statistical source data.
Source Data Extended Data Fig. 2 Statistical source data.
Source Data Extended Data Fig. 3 Uncropped western blot.
Source Data Extended Data Fig. 4 Uncropped western blot.
Source Data Extended Data Fig. 4 Statistical source data.
Source Data Extended Data Fig. 5 Uncropped western blot.
Source Data Extended Data Fig. 5 Statistical source data.
Source Data Extended Data Fig. 6 Uncropped western blot.
Source Data Extended Data Fig. 6 Statistical source data.
Source Data Extended Data Fig. 7 Uncropped western blot.
Source Data Extended Data Fig. 7 Statistical source data.
Source Data Extended Data Fig. 8 Uncropped western blot.
Source Data Extended Data Fig. 8 Statistical source data.
Source Data Extended Data Fig. 9 Uncropped western blot.
Source Data Extended Data Fig. 9 Statistical source data.
Source Data Extended Data Fig. 10 Uncropped western blot.
Source Data Extended Data Fig. 10 Statistical source data.
