# Supplementary material for: Peroxisomal β-oxidation acts as a sensor for intracellular fatty acids and regulates lipolysis
Source: Nat Metab. 2021 Dec 13;3(12):1648–61. doi: 10.1038/s42255-021-00489-2 (PMC8688145; doi:10.1038/s42255-021-00489-2)
Supplement: Supplementary file 1 — Supplementary Tables 1–3 [file 42255_2021_489_MOESM1_ESM.pdf]

nature portfolio

https://doi.org/10.1038/s42255-021-00489-2

Supplementary information

Peroxisomal β-oxidation acts as a sensor for intracellular fatty acids and regulates lipolysis

In the format provided by the authors and unedited

Table S1: SiRNA sequences used in this study.

Gene symbol	Species	Sense siRNA sequence
Pex1	Mouse	#1 GCUGCAGAAUCAAGCUAUA<br>#2 CCUUGUUCCCAUGUGGUAU<br>#3 GCAGAAGAUCUCAGUACAA
Pex2	Mouse	#1 GCUUGUGUGGUCCCAGUUU<br>#2 GCCAAACAGUUCAUGAAUU<br>#3 CCACAAUGCCUCACACUAU
Pex3	Mouse	#1 GCCUUCAAACAAGCUGGAA<br>#2 CCAGAUGAGGAGACACCAU<br>#3 GCCUGCCUUUAGCUAAGAU
Pex5	Mouse	#1 GGACCAGAAUGCAACUCUU<br>#2 GCACACAGCCAGUGACUUU<br>#3 CCUAAGCACAUGGAAGCUU
Pex6	Mouse	#1 CCUGGAGGCCUUGGUAAAU<br>#2 CCUUAGGCCAGGAGGUUAA<br>#3 GCGCAUCCAACGCAAGUUU
Pex7	Mouse	#1 CCAGCACAUCAGACGGAAA<br>#2 GCUGUGACUGGUGUAAAUA<br>#3 CCCUGUUUGUCUCACGGUU
Pex10	Mouse	#1 CAGCGCAGAAGGACGAGUA<br>#2 GCUGCAAGCCGAUGGCGAU<br>#3 GCAAAGAAAUGGCUGGAAU
Pex11a	Mouse	#1 GCACGCAUGCAUGUUGCUU<br>#2 GCGAAGAGUGUGGGUCUUA<br>#3 GGAGGUCUCAUAUCCUCUU
Pex11b	Mouse	#1 CCAGUAUGCCUGUUCCCUU<br>#2 CCAUGCUUUGCAGAGACAU<br>#3 GCUGGAUGUGCUCAGAAAU
Pex11g	Mouse	#1 GGACUGUCUUGAGACUCUU<br>#2 CCAUGUUUGUCUACACUAA<br>#3 CCCUGUGGACAAUGCUGAA
Pex12	Mouse	#1 CCCAUUACAGGGUAUCCAA<br>#2 CCAAGAAGCCAUCAAAUCA<br>#3 UCGGUUAGCUCGACUGACA
Pex13	Mouse	#1 GCAUUUCAGUCCAUUGAAA<br>#2 GCUGGUGAUAUGCUAAAUU<br>#3 GCUAGUCUUGACGGUCAAA
Pex14	Mouse	#1 CCAGGAGAGCAUUUCUUAA<br>#2 GGCGAGAUUAUGGUGCCUU<br>#3 CCUGGAGUCCCAGAAUAUU
Pex16	Mouse	#1 GGAGUAUGUGACUCGUCAU<br>#2 GAUCCUGAGGAAGGAGCUU

		#3 GCUUACAGACCACAUCCCU
Pex19	Mouse	#1 GCUUCUGGAAAGUGCUCUU<br>#2 GCCAGGAGAUACUGCCAAA<br>#3 GCGAACAGUGUCUGAUCAU
Pex26	Mouse	#1 GGCUCUGGCAGAAAUGGAU<br>#2 CCAGGGCCUUCCUGAUUAU<br>#3 CCUCUUGAUUCUACGGUUU
Acox1	Mouse	#1 GUUACGAGGUGGCUGUUAA<br>#2 GUAACAAGCUGACAUAUGG
Cat	Mouse	#1 CCAGAUACUCCAAGGCAAA<br>#2 GGAAACGCCUGUGUGAGAA
Atgl	Mouse	#1 AGGGAGACCAAGUGGAACA<br>#2 GGAAAUUGGGUGACCAUCU<br>#3 GCACAUUUAUCCCGGUGUA
Cpt1b	Mouse	#1 AGAAAUACCUGGUGCUCAA
Cpt2	Mouse	#1 CCUCCAGCAAGAAAAUAUC
Cop1	Mouse	#1 GGGCAUUGGGUCACGCAAA<br>#2 CUACAAGGAUGUCUCGUAU<br>#3 UGUCCAAGUUUACUCGAUA<br>#4 ACAUUAGCAUCAAGACGAA
PEX2	Human	#1 GCUAGUUUGGUCCCAGUUU<br>#2 GGUAUGCUGUUUGUACAAU<br>#3 GAAGAACGAUGCUAUGAUU
PEX10	Human	#1 GGUUGAGCUGCUCUCAGAU
PEX12	Human	#1 GCAGCUUUGGAAAUCUAUU
PEX5	Human	#1 CGUAUCCUGGGAUCUCUCU
PEX19	Human	#1 GCUUCUGGAAAGUGCUCUU
CPT1A	Human	#1 GCCUCUUAUGAAGGAAGAA
CPT2	Human	#1 GCUGGUUGCUCUGGACAAA
ACOX1	Human	#1 GGAAAGACUUCAAAUCAUG<br>#2 UUACAUGCCUUUAUCGUAC
CAT	Human	#1 CCAAAUACUCCAAGGCAAA<br>#2 CCAGGUCAUGACAUUUAAU
ATGL	Human	#1 GCGAGAAGACGUGGAACAU<br>#2 CCUGCCACUCUAUGAGCUU<br>#3 GCACCUGUGCCUUAAUCUU

Table S2: Sequences of qPCR primers used in this study.

Gene symbol	Species	Primer set for qPCR
Pex2	Mouse	F: aaaaatgattcttctctcaacctga<br>R: tgcacacagcataccacagtt
Pex10	Mouse	F: aagagactagcagggatcacgta<br>R: gtagctagtgcgagccttctg
Pex12	Mouse	F: cgctcttcagcatgtggtc<br>R: ctggagcaggaagtctagcag
Pparg2	Mouse	F: gcatggtgccttcgctga<br>R: tggcatctctgtgtcaaccatg
Adipoq	Mouse	F: cgattgtcagtggatctgacg<br>R: caacagtagcatcctgagccct
Atgl	Mouse	F: tgaccatctgccttccaga<br>R: tgtaggtggcgcaagaca
36B4	Mouse	F: gccgtgatgcccagggaaga<br>R: catctgcttggagcccacgtt
PEX2	Human	F: catggtgtattcctcttactggtg<br>R: ggccactctccacatagagc
PEX10	Human	F: tttccagaaaccccctgtg<br>R: gatgcactcccagcagaac
PEX12	Human	F: aaccagctaaggccagcat<br>R: cagctttcttcagagctgagttta
ATGL	Human	F: ctccaccaacatccacgag<br>R: ccctgcttgcacatctctc
36B4	Human	F: ccaggcgtcctcgtggaagt<br>R: tgctgcatctgcttggagccca

Table S3: Clinical information for NAFLD patients

Abbreviations: female (f), male (m), not measured (N/A)

Biopsy_ID	Steatosis (%)	Age at Biopsy	Sex	BMI kg/m2	ballooning	inflammation
1	10	36	f	39	1	0
2	15	27	m	N/A	N/A	0
3	20	63	f	31	N/A	1
4	20	61	f	29	0	0
5	20	49	m	28	0	0
6	20	52	m	N/A	0	1
7	30	42	m	30	1	1
8	30	62	m	38	0	1
9	30	37	f	35	0	1
10	40	49	m	26	N/A	1
11	40	54	m	28	1	0
12	40	32	f	40	0	0
13	40	34	m	N/A	0	1
14	40	34	m	28	N/A	1
15	60	46	f	30	0	1
16	70	50	f	N/A	N/A	N/A
17	75	62	m	N/A	N/A	1
18	80	43	m	N/A	N/A	1
19	80	51	m	31	0	1
20	80	74	f	N/A	N/A	1
21	80	42	f	35	0	1
22	90	58	f	N/A	0	1
23	90	22	f	21	1	1
24	95	51	m	32	N/A	1
25	20	20	m	49	0	0
26	5	25	m	56	0	0
27	10	34	m	51	0	0
28	0	26	m	63	0	0
29	25	24	m	41	0	0
30	10	47	m	57	0	0
31	30	41	m	40	0	0
32	0	23	m	63	0	0
33	15	38	m	46	0	0
34	3	42	m	43	0	0
35	30	24	m	49	0	0
36	60	43	m	56	0	0
37	60	47	m	43	0	0
38	35	45	m	49	0	0
39	50	23	m	45	0	0
40	50	27	m	49	0	0
41	40	49	m	38.2	0	0
42	30	26	m	43.4	0	0
43	80	44	m	40	0	0
44	90	43	m	47.4	0	0
45	70	35	m	41.5	0	0
46	70	37	m	44.1	0	0
47	70	37	m	53.1	0	0
48	70	37	m	54.9	0	0
49	20	49	m	42.5	0	0
50	70	26	m	53.6	0	0
