# Supplementary material for: Peroxisomal β-oxidation acts as a sensor for intracellular fatty acids and regulates lipolysis
Source: Nat Metab. 2021 Dec 13;3(12):1648–61. doi: 10.1038/s42255-021-00489-2 (PMC8688145; doi:10.1038/s42255-021-00489-2)
Supplement: Supplementary file 2 — Reporting Summary [file 42255_2021_489_MOESM2_ESM.pdf]

nature research

Corresponding author(s): Christian Wolfrum

Last updated by author(s): Sep 25, 2021

Reporting Summary

Nature Research wishes to improve the reproducibility of the work that we publish. This form provides structure for consistency and transparency in reporting. For further information on Nature Research policies, see our Editorial Policies and the Editorial Policy Checklist.

Statistics

For all statistical analyses, confirm that the following items are present in the figure legend, table legend, main text, or Methods section.

n/a	Confirmed	
☐	☒	The exact sample size (*n*) for each experimental group/condition, given as a discrete number and unit of measurement
☐	☒	A statement on whether measurements were taken from distinct samples or whether the same sample was measured repeatedly
☐	☒	The statistical test(s) used AND whether they are one- or two-sided<br>*Only common tests should be described solely by name; describe more complex techniques in the Methods section.*
☐	☒	A description of all covariates tested
☐	☒	A description of any assumptions or corrections, such as tests of normality and adjustment for multiple comparisons
☐	☒	A full description of the statistical parameters including central tendency (e.g. means) or other basic estimates (e.g. regression coefficient) AND variation (e.g. standard deviation) or associated estimates of uncertainty (e.g. confidence intervals)
☐	☒	For null hypothesis testing, the test statistic (e.g. *F*, *t*, *r*) with confidence intervals, effect sizes, degrees of freedom and *P* value noted<br>*Give P values as exact values whenever suitable.*
☒	☐	For Bayesian analysis, information on the choice of priors and Markov chain Monte Carlo settings
☒	☐	For hierarchical and complex designs, identification of the appropriate level for tests and full reporting of outcomes
☒	☐	Estimates of effect sizes (e.g. Cohen's *d*, Pearson's *r*), indicating how they were calculated

Our web collection on statistics for biologists contains articles on many of the points above.

Software and code

Policy information about availability of computer code

Data collection	No specific software was used
Data analysis	ImageJ (version 1.53e) was used to analyze western blots and images. Graphpad prism (version 9.1) was used to perform statistical analyses. Harmony 3.5 was used in high content adipocyte differentiation determination

For manuscripts utilizing custom algorithms or software that are central to the research but not yet described in published literature, software must be made available to editors and reviewers. We strongly encourage code deposition in a community repository (e.g. GitHub). See the Nature Research guidelines for submitting code & software for further information.

Data

Policy information about availability of data

All manuscripts must include a data availability statement. This statement should provide the following information, where applicable:

- Accession codes, unique identifiers, or web links for publicly available datasets
- A list of figures that have associated raw data
- A description of any restrictions on data availability

All raw data are available

Field-specific reporting

Please select the one below that is the best fit for your research. If you are not sure, read the appropriate sections before making your selection.

☒ Life sciences ☐ Behavioural & social sciences ☐ Ecological, evolutionary & environmental sciences

For a reference copy of the document with all sections, see nature.com/documents/nr-reporting-summary-flat.pdf

Life sciences study design

All studies must disclose on these points even when the disclosure is negative.

Sample size
Sample sizes were determined on the basis of previous experiments using similar methods (ref. 39).

Data exclusions
No samples were excluded from any analyses.

Replication
Experimental findings were verified by biological replicates and technical replicates, each experiment was performed multiple times as indicated in the figure legends.

Randomization
All experiments were randomized

Blinding
The investigators were not blinded to the mice as they themselves were treating and sacrificing the mice. However, the investigators were blinded for sample processing.

Reporting for specific materials, systems and methods

We require information from authors about some types of materials, experimental systems and methods used in many studies. Here, indicate whether each material, system or method listed is relevant to your study. If you are not sure if a list item applies to your research, read the appropriate section before selecting a response.

Materials & experimental systems

n/a	Involved in the study	
☐	☒	Antibodies
☐	☒	Eukaryotic cell lines
☒	☐	Palaeontology and archaeology
☐	☒	Animals and other organisms
☐	☒	Human research participants
☐	☒	Clinical data
☒	☐	Dual use research of concern

Methods

n/a	Involved in the study	
☒	☐	ChIP-seq
☒	☐	Flow cytometry
☒	☐	MRI-based neuroimaging

Antibodies

Antibodies used
ATGL (1:1000, Cell Signaling:2138S), CGI-58 (1:1000, Proteintech:12201-1-AP), phosphor-HSL(Ser660) (1:1000, Cell Signaling:45804S), PLIN1 (1:1000, Cell Signaling:9349S), HSL (1:1000, Cell Signaling:4107S), γ-tubulin (1:10000, Sigma:T6557), FLAG (1:10000, Sigma:F3165; 1:1000, Cell Signaling:2368S), PEX10 (1:1000, Sigma:WH0005192M1), PEX12 (1:1000, Abcam:ab103456), CAT (1:1000, Cell Signaling:14097S), ACOX1 (1:1000, Abcam:ab184032), HSP90 (1:1000, Cell Signaling:4877S), K48 or K63 linkage specific ubiquitin antibody (1:1000, Cell Signaling:33959), HA (1:1000, Cell signaling:3724S), Myc (1:1000, Sigma:C3956), PMP70 antibody (1;10000, Sigma:SAB4200181), PEX2(1:1000, Thermo:PA5-26187), Calnexin (1:1000, Cell signaling:2433S), EGFP(1:1000, Abcam:ab290), Cysteine sulfenic acid (1:1000, Sigma:ABS30), p-mTORSer2448(1:1000, Cell signaling:2974S), p-S6KThr389(1:1000, Cell signaling:9205S), S6K(1:1000, Cell signaling:9202S), LC3 (1:1000, Cell signaling:2775S), COP1(1:1000, abcam:ab56400), HRP-conjugated anti-rabbit IgG (1:10000, Cell signaling: 7074), HRP-conjugated anti-mouse IgG (1:10000, Cell signaling: 7076),Goat anti-Rabbit IgG (H+L) Cross-Adsorbed Secondary Antibody, Alexa Fluor 568 (1:200, Thermo:A11011),Donkey anti-Mouse IgG (H+L) Highly Cross-Adsorbed Secondary Antibody, Alexa Fluor 488 (1:200, Thermo:A21202)

Validation
We validated ATGL,ACOX1 and CAT antibodies in both mouse and human cell lines for WB application via siRNA knockdown.
We validated PEX10, PEX12 antibodies in human cell line for WB application via siRNA knockdown.
We validated COP1 antibodies in mouse cell line for WB application via siRNA knockdown.
The following antibodies were validated by previous publications, ATGL(10.7554/eLife.63665),CGI-58 (10.1038/s42255-019-0066-3), PLIN1 (10.1172/jci.insight.139160), HSL (10.1172/jci.insight.139160), K48 or K63 linkage specific ubiquitin antibody (10.1038/s41419-020-03299-8), PMP70 (10.1038/ncb3230), Calnexin (10.1111/acel.13368), HSP90 (10.1371/journal.ppat.1009582),p-mTORSer2448, LC3 (https://doi.org/10.1016/j.molcel.2020.05.007), p-mTORSer2448 (https://doi.org/10.1016/j.molcel.2020.05.007), S6K (https://doi.org/10.1016/j.molcel.2020.05.007), p-S6KThr389 (https://doi.org/10.1016/j.molcel.2020.05.007), Cysteine sulfenic acid (10.1371/journal.pone.0180455),EGFP (10.1080/15548627.2020.1740529), Myc (10.1016/j.cell.2008.08.036), HA (10.1038/s41467-021-24097-6)

Eukaryotic cell lines

Policy information about cell lines

Cell line source(s)	293-AAV and 293-LTV cell lines were purchased from Cell Biolabs Inc. (cat. AAV-100; LTV-100); HEK293T cell line was purchased from abcam. iBAs cells were previously established by Ronald Kahn lab as indicated in manuscript. HepG2 cells were purchased from ATCC.
Authentication	293-AAV and 293-LTV cells were not authenticated, however the AAV and LV produced from these cells were titred by PCR. iBA cells were previously validate by gene expression and functional assay (OCR). HepG2 cells and HEK293Tcells were validated via PCR.
Mycoplasma contamination	The cells were regularly tested, all cell lines tested were negative for mycoplasma contamination.
Commonly misidentified lines (See ICLAC register)	No commonly misidentified cell lines were used in the study.

Animals and other organisms

Policy information about studies involving animals; ARRIVE guidelines recommended for reporting animal research

Laboratory animals	C57Bl/6 female mice were used. B6J.129(B6N)-Gt(ROSA)26Sortm1(CAG-cas9*,-EGFP)Fezh/J, Acox1 floxed and Atgl floxed female mice were used. All the experiments were started when mice were 8 weeks of age.
Wild animals	No wild animals were used in the study.
Field-collected samples	No field collected samples were used in the study.
Ethics oversight	All experiments were approved by the Animal Ethics Committee of Zurich, all animal studies were approved by the Veterinäramt Zürich.

Note that full information on the approval of the study protocol must also be provided in the manuscript.

Human research participants

Policy information about studies involving human research participants

Population characteristics	Patients with liver diseases or severe obesity,above 20 years, at both genders, were adults of European (92%) and/or African (8%) ancestry with different medical treatment
Recruitment	Human liver biopsies were collected in two cohorts and were analyzed for data integration. Liver biopsies of one cohort were obtained during the work-up of liver disease diagnostics in the outpatient clinic of the Division of Gastroenterology and Hepatology, University Hospital Basel, Switzerland. The study was carried out in accordance with The Code of Ethics of the World Medical Association (Declaration of Helsinki) and was approved by the Ethics Committee of North Western Switzerland (Authorization number EKNZ 2014-362). Written informed consent was obtained from all patients enrolled in this study. Five to ten millimeters of the liver biopsy cylinder were immediately snap frozen by immersion in liquid nitrogen and stored in liquid nitrogen vapors until processing and analysis.<br>Human liver biopsies of the other cohort were obtained from participants of the Biological Atlas of Severe Obesity (ABOS) cohort (ClinicalTrials.gov identifier NCT01129297), an ongoing prospective cohort study for the longitudinal assessment of metabolic outcomes after weight loss surgery. The study design has been described previously in details44. Briefly, participants were adults of European (92%) and/or African (8%) ancestry, who fulfilled the criteria for weight loss surgery, including severe obesity [body mass index (BMI) ≥40 kg/m2 or ≥ 35 kg/m2 with comorbidities] for at least 5 years and resistance to medical treatment, and the absence of medical or psychological contraindications to surgery. Patients with current excessive drinking (daily consumption of alcohol ≥20 g/day for women and ≥ 30 g/day for men), history of past excessive drinking for a period longer than 2 years at any time in the past 20 years, long-term consumption of hepatotoxic drugs, or positive screening for chronic liver diseases including positive testing for hepatitis B surface antigen and hepatitis C virus antibodies, evidence of genetic hemochromatosis, and age < 20 years were excluded. All enrolled patients gave their informed consent for a comprehensive metabolic phenotyping with tissue, plasma and serum sampling prior to the intervention.
Ethics oversight	Cohort of Basel group was carried out in accordance with The Code of Ethics of the World Medical Association (Declaration of Helsinki) and was approved by the Ethics Committee of North Western Switzerland (Authorization number EKNZ 2014-362). Biological Atlas of Severe Obesity (ABOS) cohort was approved with ClinicalTrials.gov identifier NCT01129297 and All procedures were approved by the C.H.R.U. Lille Ethical committee and were compliant to the French National Ethics Committee guidelines

Note that full information on the approval of the study protocol must also be provided in the manuscript.

Clinical data

Policy information about clinical studies
All manuscripts should comply with the ICMJE guidelines for publication of clinical research and a completed CONSORT checklist must be included with all submissions.

Clinical trial registration	EKNZ 2014-362 and NCT01129297
Study protocol	Protocol of Basel cohort:Liver biopsies of one cohort were obtained during the work-up of liver disease diagnostics in the outpatient clinic of the Division of Gastroenterology and Hepatology, University Hospital Basel, Switzerland. Five to ten millimeters of the liver biopsy cylinder were immediately snap frozen by immersion in liquid nitrogen and stored in liquid nitrogen vapors until processing and analysis. The full protocol is described in previous publication (doi.org/10.1016/j.jhep.2021.04.051)<br>Protocol of French cohort:participants were adults of European (92%) and/or African (8%) ancestry, who fulfilled the criteria for weight loss surgery, including severe obesity [body mass index (BMI) ≥40 kg/m2 or ≥ 35 kg/m2 with comorbidities] for at least 5 years and resistance to medical treatment, and the absence of medical or psychological contraindications to surgery. Patients with current excessive drinking (daily consumption of alcohol ≥20 g/day for women and ≥ 30 g/day for men), history of past excessive drinking for a period longer than 2 years at any time in the past 20 years, long-term consumption of hepatotoxic drugs, or positive screening for chronic liver diseases including positive testing for hepatitis B surface antigen and hepatitis C virus antibodies, evidence of genetic hemochromatosis, and age < 20 years were excluded. All enrolled patients gave their informed consent for a comprehensive metabolic phenotyping with tissue, plasma and serum sampling prior to the intervention.<br>Liver biopsy was systematically planned during the surgical procedure and performed during the first part of the surgical procedure after trocar insertion and abdominal exploration, within 10 minutes after pneumo-peritoneum installation. A wedge liver surgical biopsy was performed, fragmented in 30 mg samples, and snap frozen in liquid nitrogen and stored at -80°C. In addition, a needle biopsy was performed for histological assessment of steatosis. Following staining with H&E saffron, Sirius red, and Perl's staining, steatosis was quantified by the percentage of hepatocytes containing fat droplets (amount of steatosis). The full protocol is described in previous publication (10.1172/JCI68815)
Data collection	First cohort: Liver biopsies of this cohort were obtained during the work-up of liver disease diagnostics in the outpatient clinic of the Division of Gastroenterology and Hepatology, University Hospital Basel, Switzerland. Five to ten millimeters of the liver biopsy cylinder were immediately snap frozen by immersion in liquid nitrogen and stored in liquid nitrogen vapors until processing and analysis to check steatosis level.<br>Second cohort: Liver biopsies of this cohort were obtained in Lille University Hospital. Liver biopsy was systematically planned during the surgical procedure and performed during the first part of the surgical procedure after trocar insertion and abdominal exploration, within 10 minutes after pneumo-peritoneum installation. A wedge liver surgical biopsy was performed, fragmented in 30 mg samples, and snap frozen in liquid nitrogen and stored at -80°C.A needle biopsy was performed for histological assessment of steatosis. Following staining with H&E saffron, Sirius red, and Perl's staining, steatosis was quantified by the percentage of hepatocytes containing fat droplets (amount of steatosis).<br>After liver biospy sampling, the samples were further homogenized in ETH, Zurich for protein measurement and data collection
Outcomes	The liver biopsies of different steatosis levels were homogenized to measure ATGL protein level and cysteine sulfenic acid modification levels and the correlation between steatosis levels and ATGL or ROS levels in the biopsies were analyzed and shown in the manuscript
