# Supplementary figures and images for: Peroxisomal β-oxidation acts as a sensor for intracellular fatty acids and regulates lipolysis
Source: Nat Metab. 2021 Dec 13;3(12):1648–61. doi: 10.1038/s42255-021-00489-2 (PMC8688145; doi:10.1038/s42255-021-00489-2)

Fig.1

c

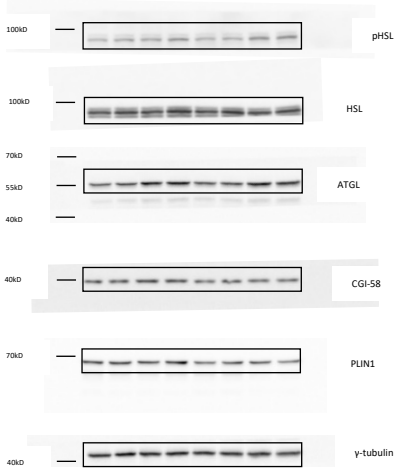

f

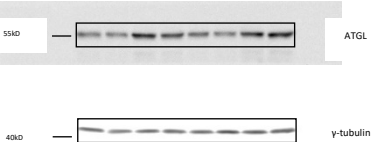

e

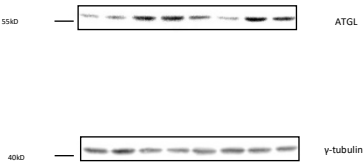

g

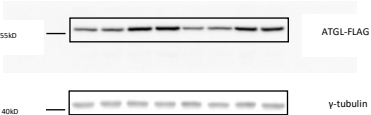

Supplement: Source Data Fig. 1 — Uncropped western blot. [file 42255_2021_489_MOESM3_ESM.pdf]

Fig.2

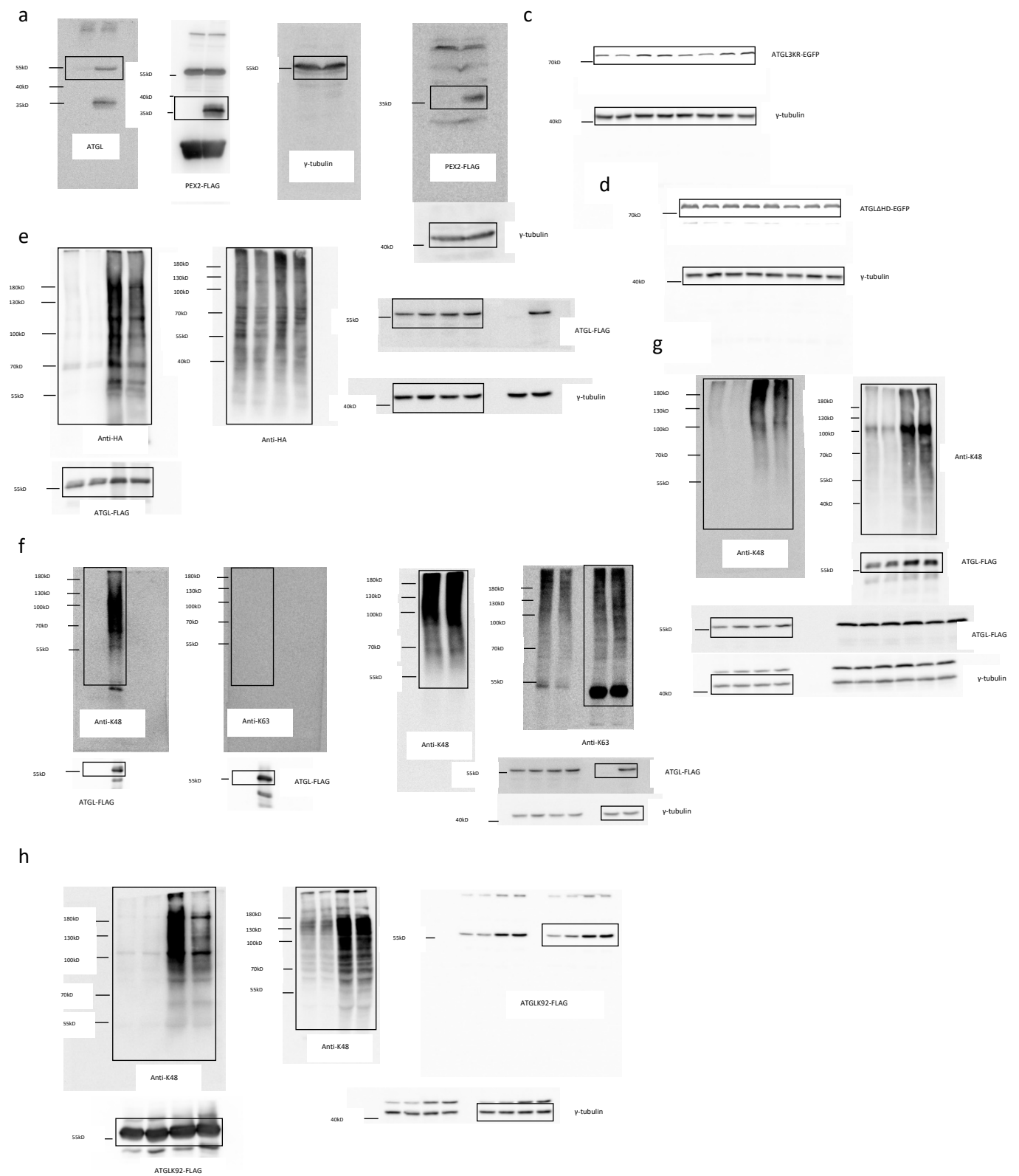

Supplement: Source Data Fig. 2 — Uncropped western blot. [file 42255_2021_489_MOESM5_ESM.pdf]

Fig.3

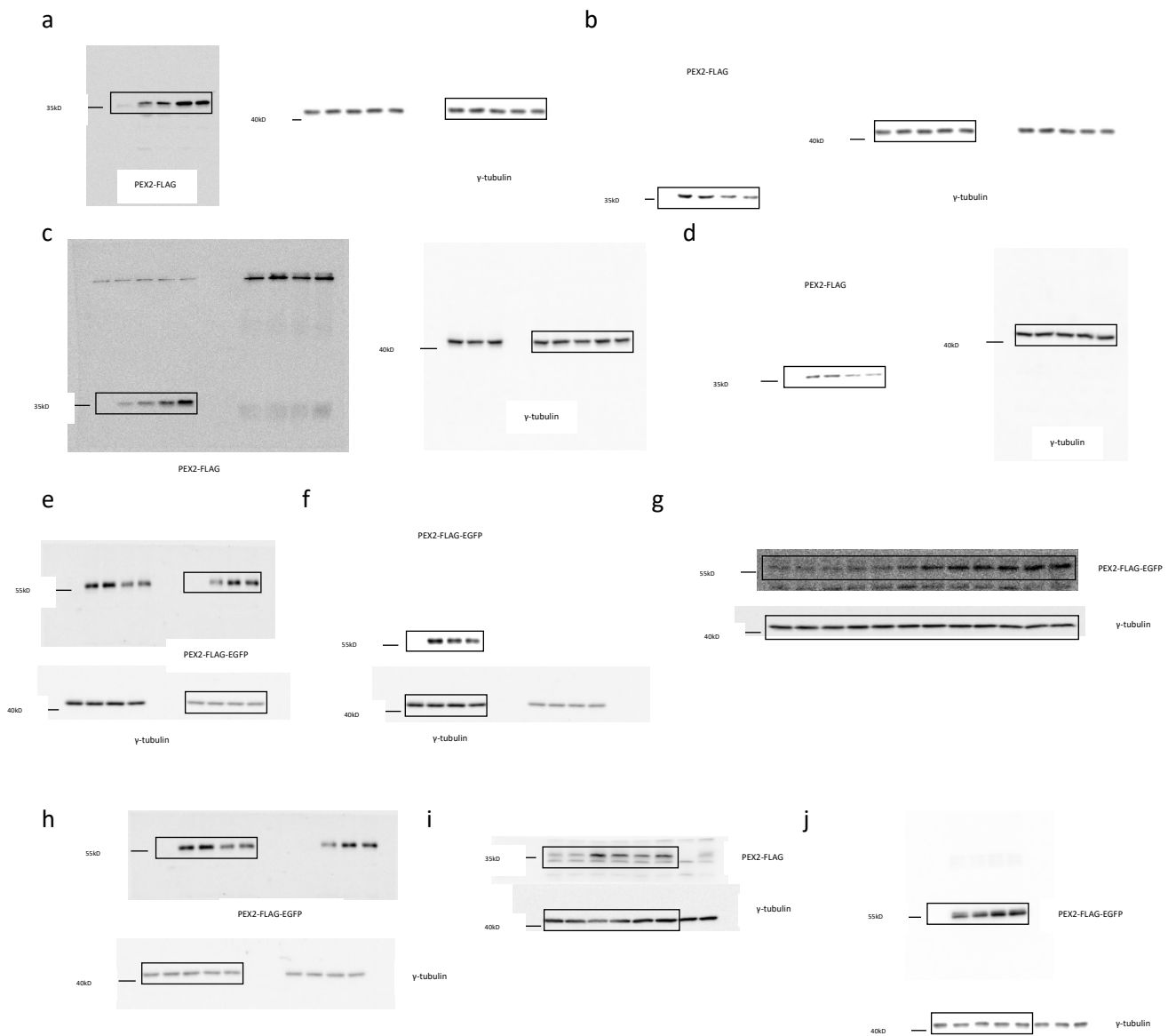

Supplement: Source Data Fig. 3 — Uncropped western blot. [file 42255_2021_489_MOESM7_ESM.pdf]

Fig.4

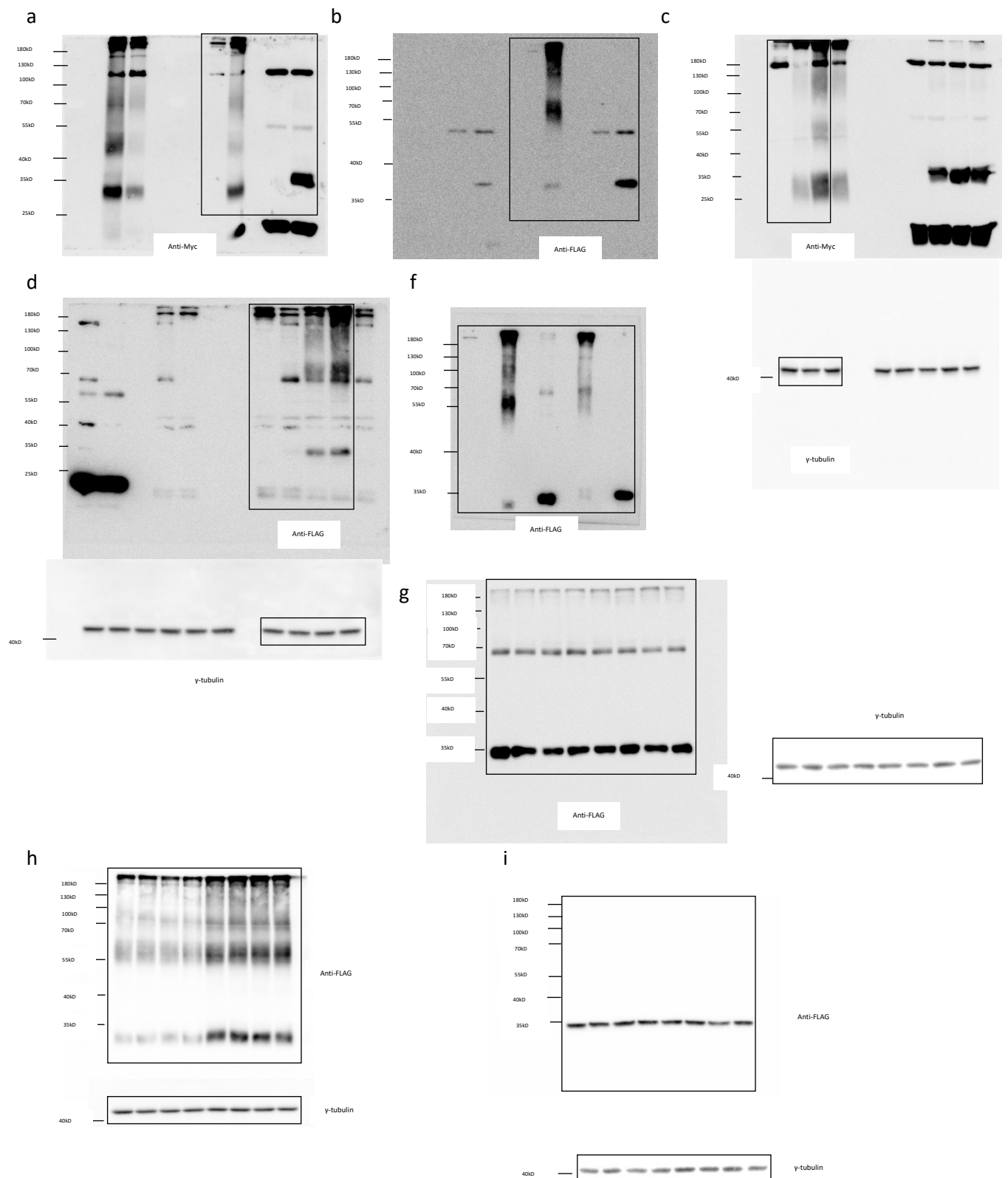

Supplement: Source Data Fig. 4 — Uncropped western blot. [file 42255_2021_489_MOESM9_ESM.pdf]

Fig.5

a

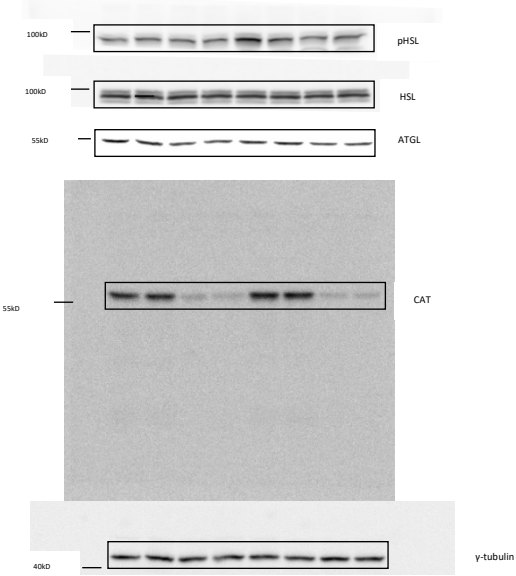

d

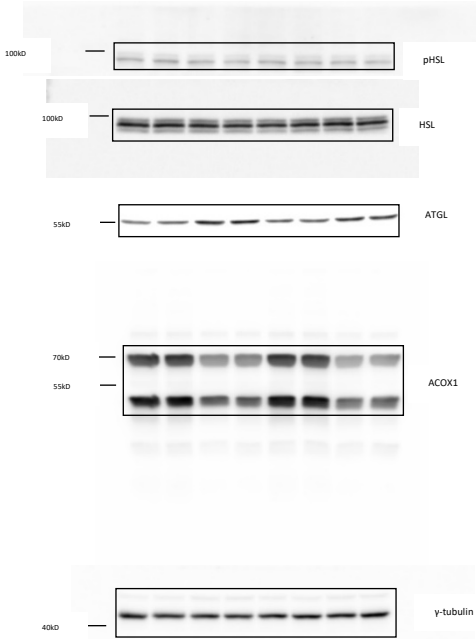

g

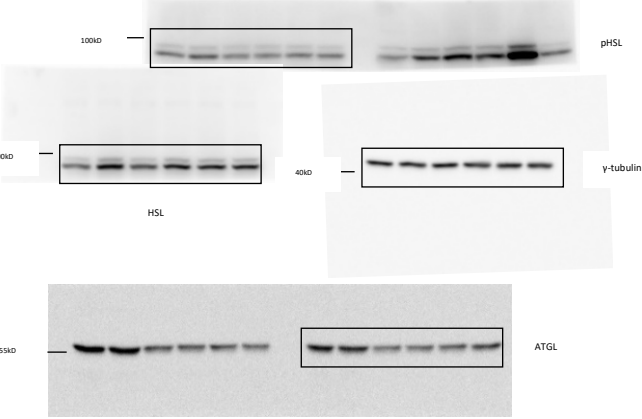

j

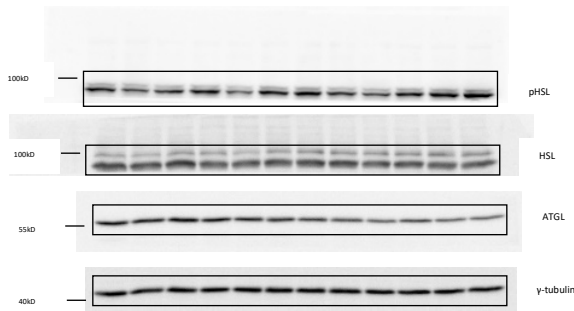

m

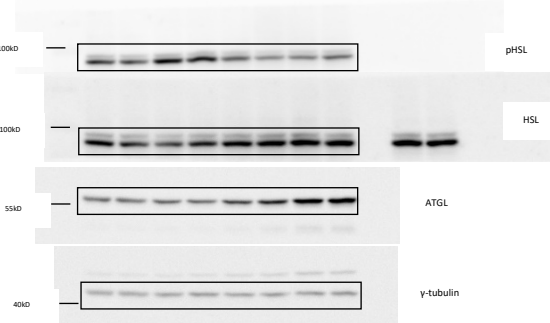

Supplement: Source Data Fig. 5 — Uncropped western blot. [file 42255_2021_489_MOESM10_ESM.pdf]

Fig.6

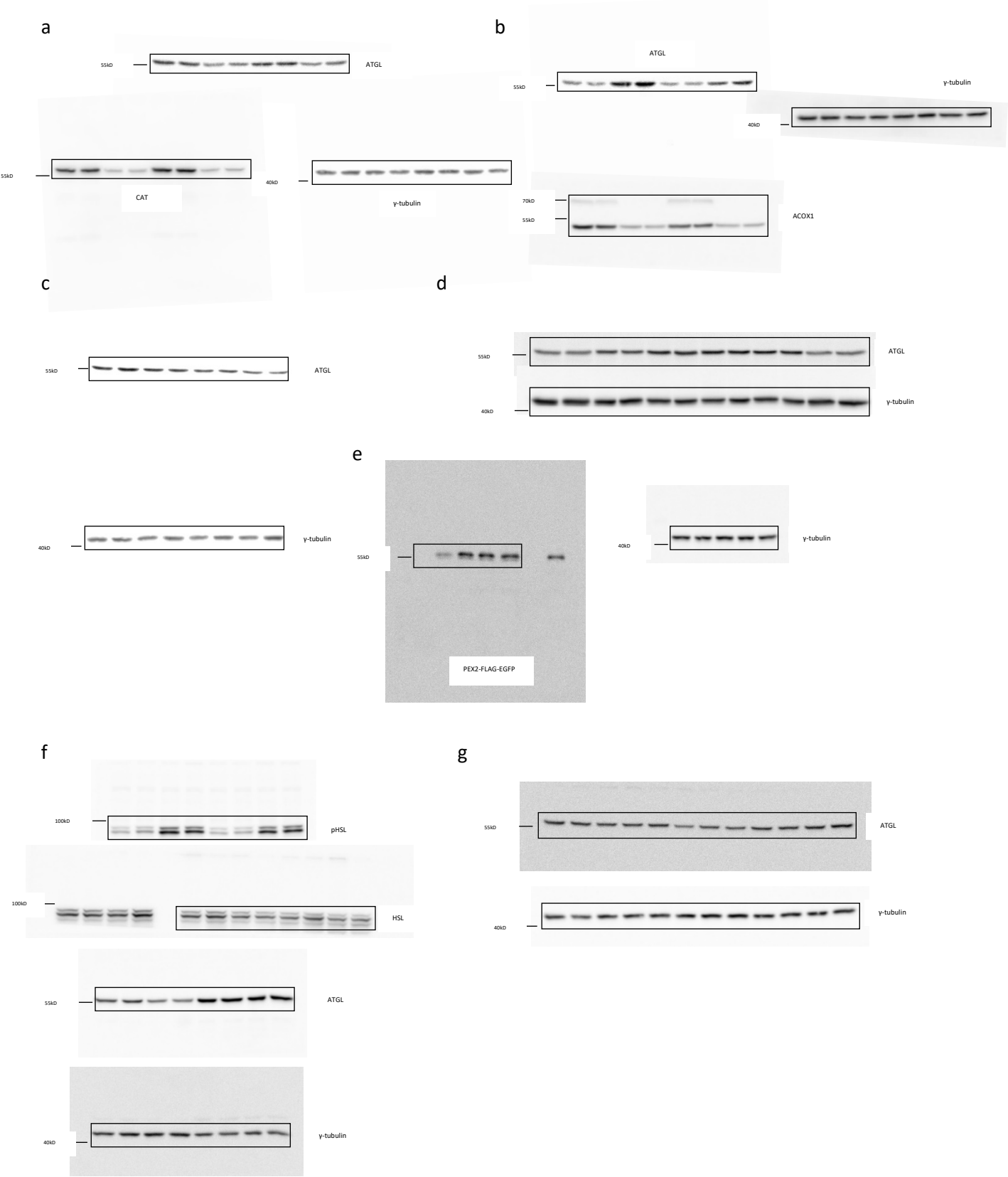

Supplement: Source Data Fig. 6 — Uncropped western blot. [file 42255_2021_489_MOESM12_ESM.pdf]

Fig.7

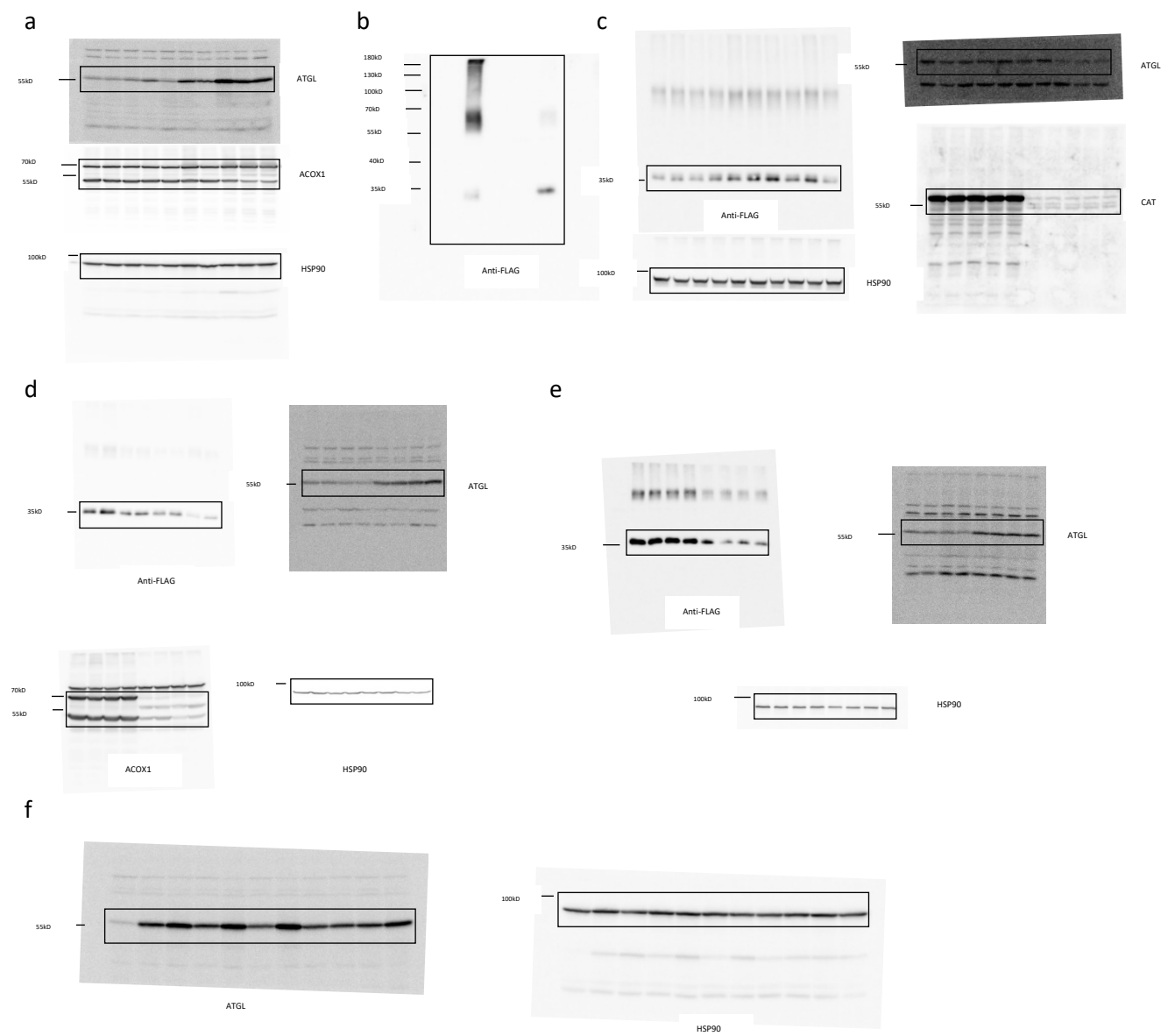

Supplement: Source Data Fig. 7 — Uncropped western blot. [file 42255_2021_489_MOESM14_ESM.pdf]

Extended Data Fig.1

k

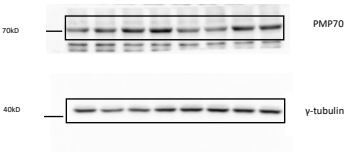

m

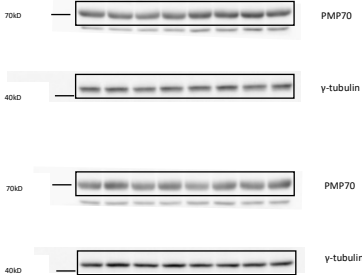

Supplement: Source Data Extended Data Fig. 1 — Uncropped western blot. [file 42255_2021_489_MOESM16_ESM.pdf]

Extended Data Fig.2

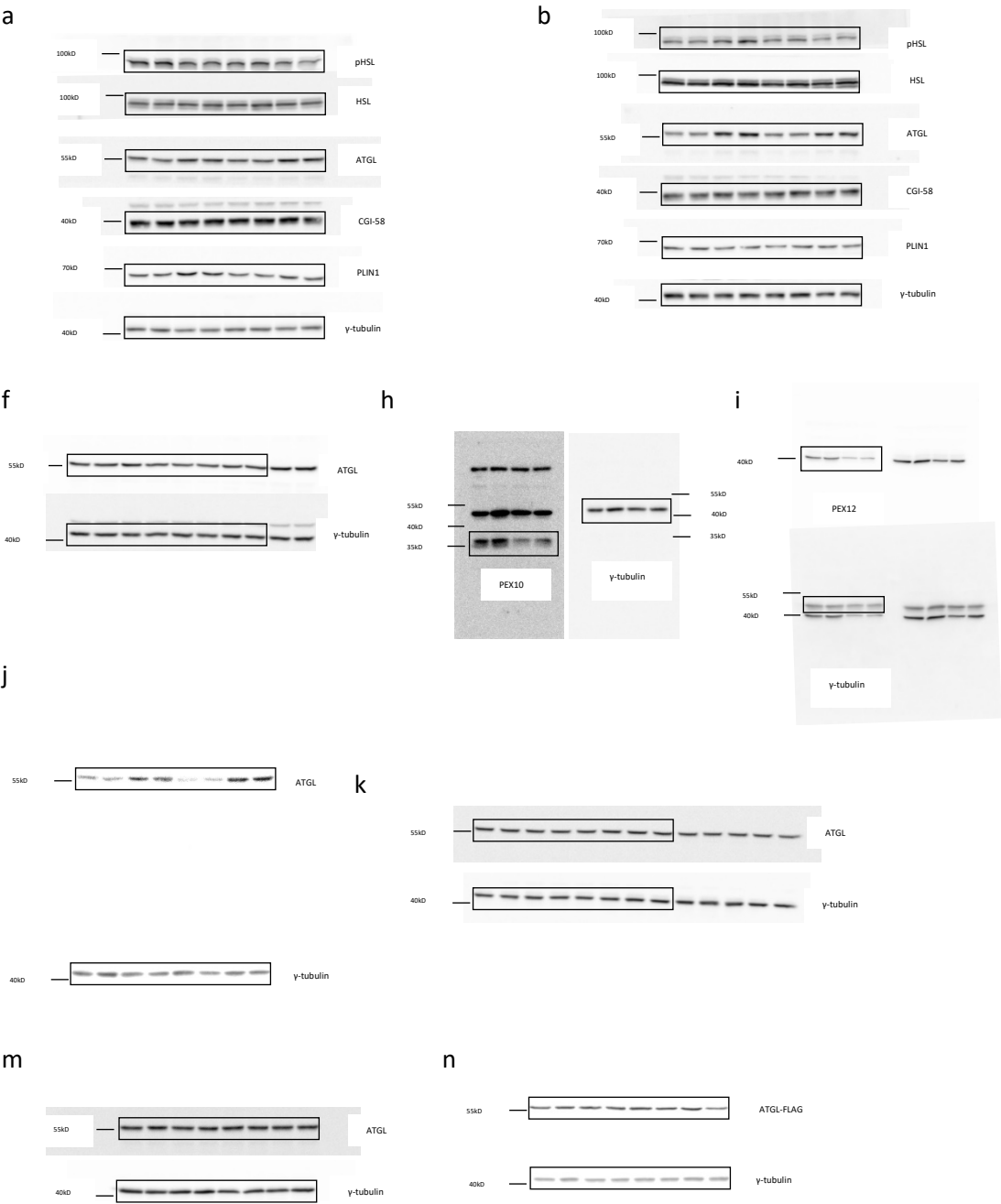

Supplement: Source Data Extended Data Fig. 2 — Uncropped western blot and statistical source data. [file 42255_2021_489_MOESM18_ESM.pdf]

Extended Data Fig.3

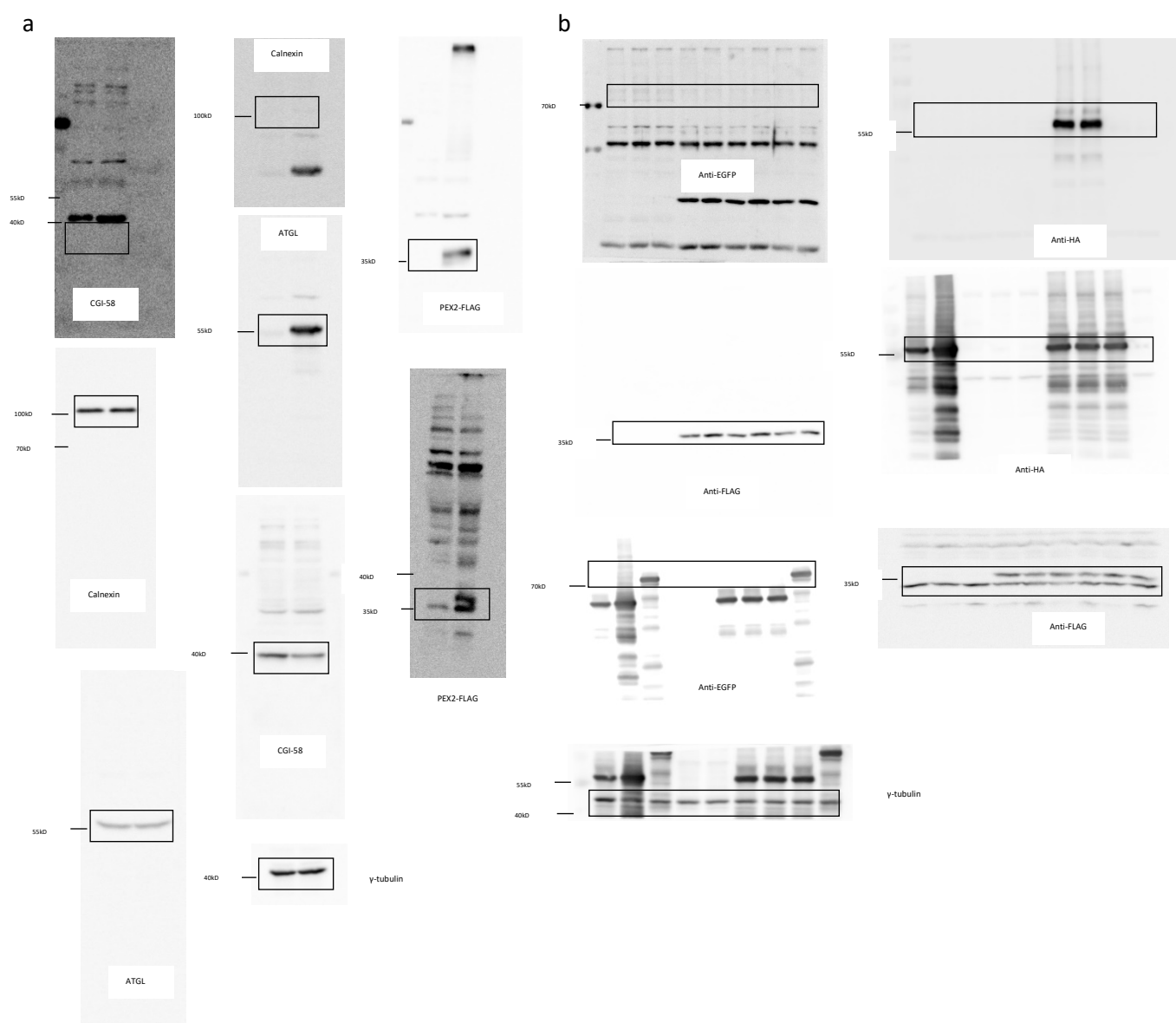

Supplement: Source Data Extended Data Fig. 3 — Uncropped western blot. [file 42255_2021_489_MOESM20_ESM.pdf]

Extended Data Fig.4

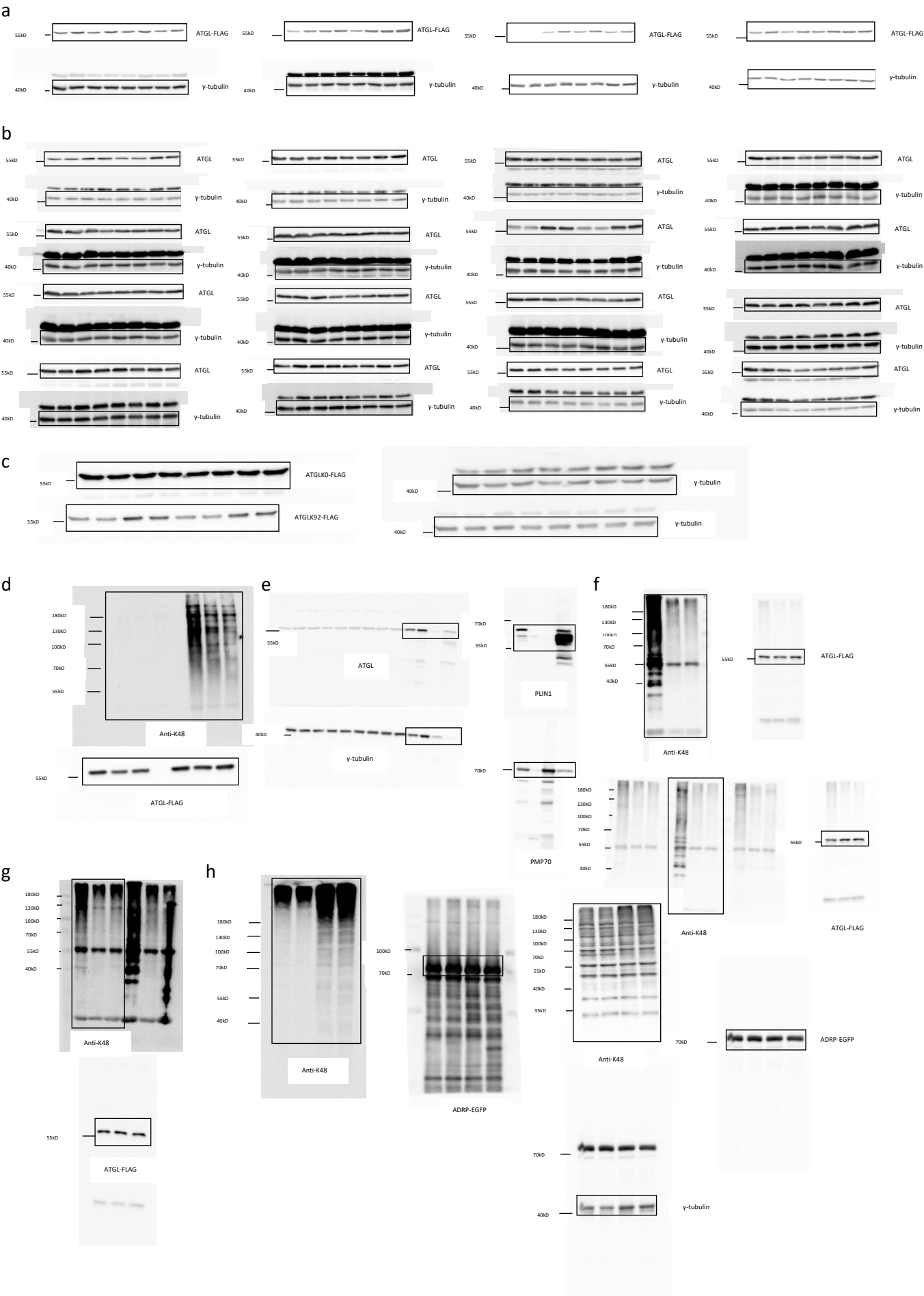

Supplement: Source Data Extended Data Fig. 4 — Uncropped western blot. [file 42255_2021_489_MOESM21_ESM.pdf]

Extended Data Fig.5

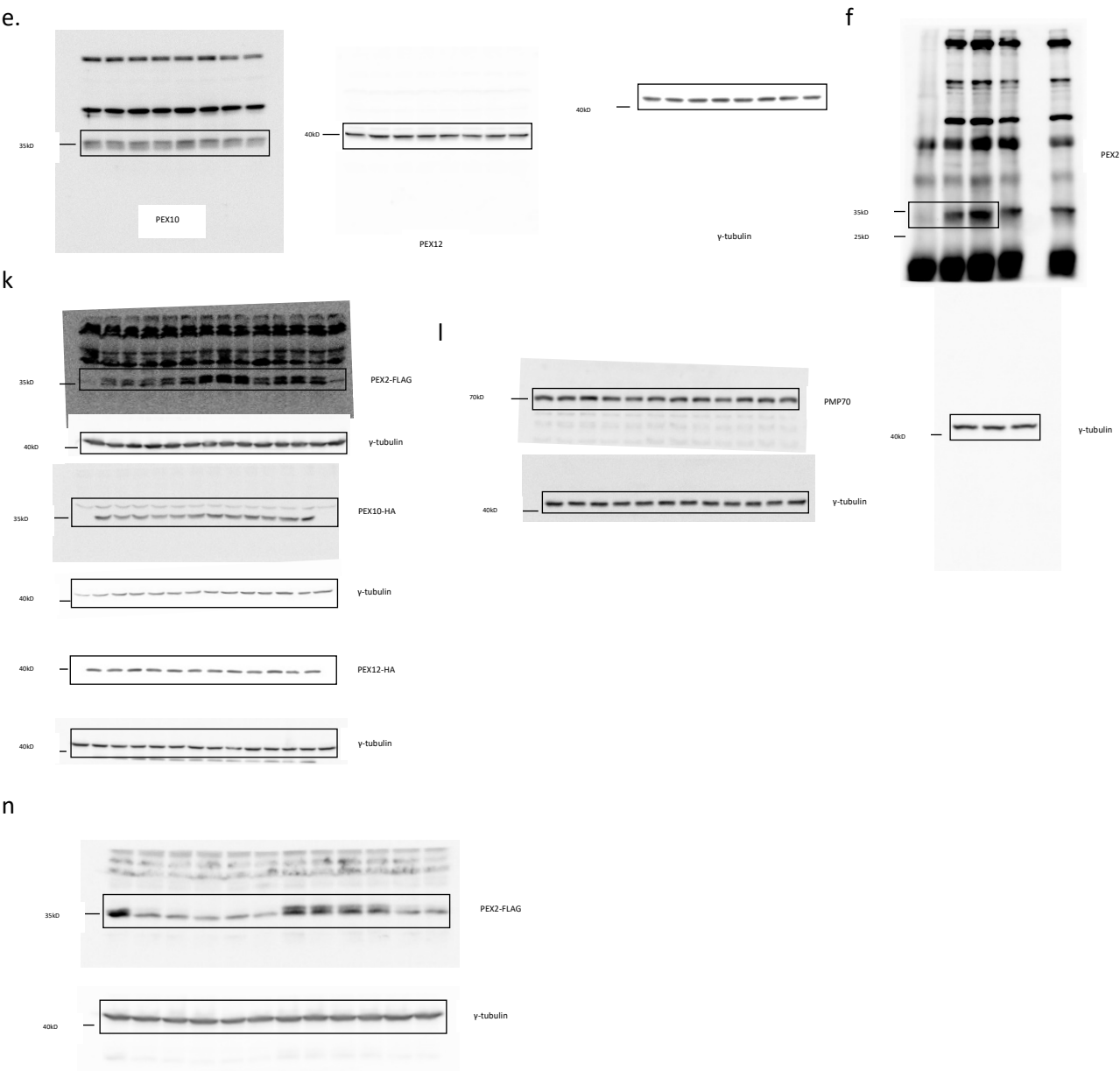

Supplement: Source Data Extended Data Fig. 5 — Uncropped western blot. [file 42255_2021_489_MOESM23_ESM.pdf]

Extended Data Fig.6

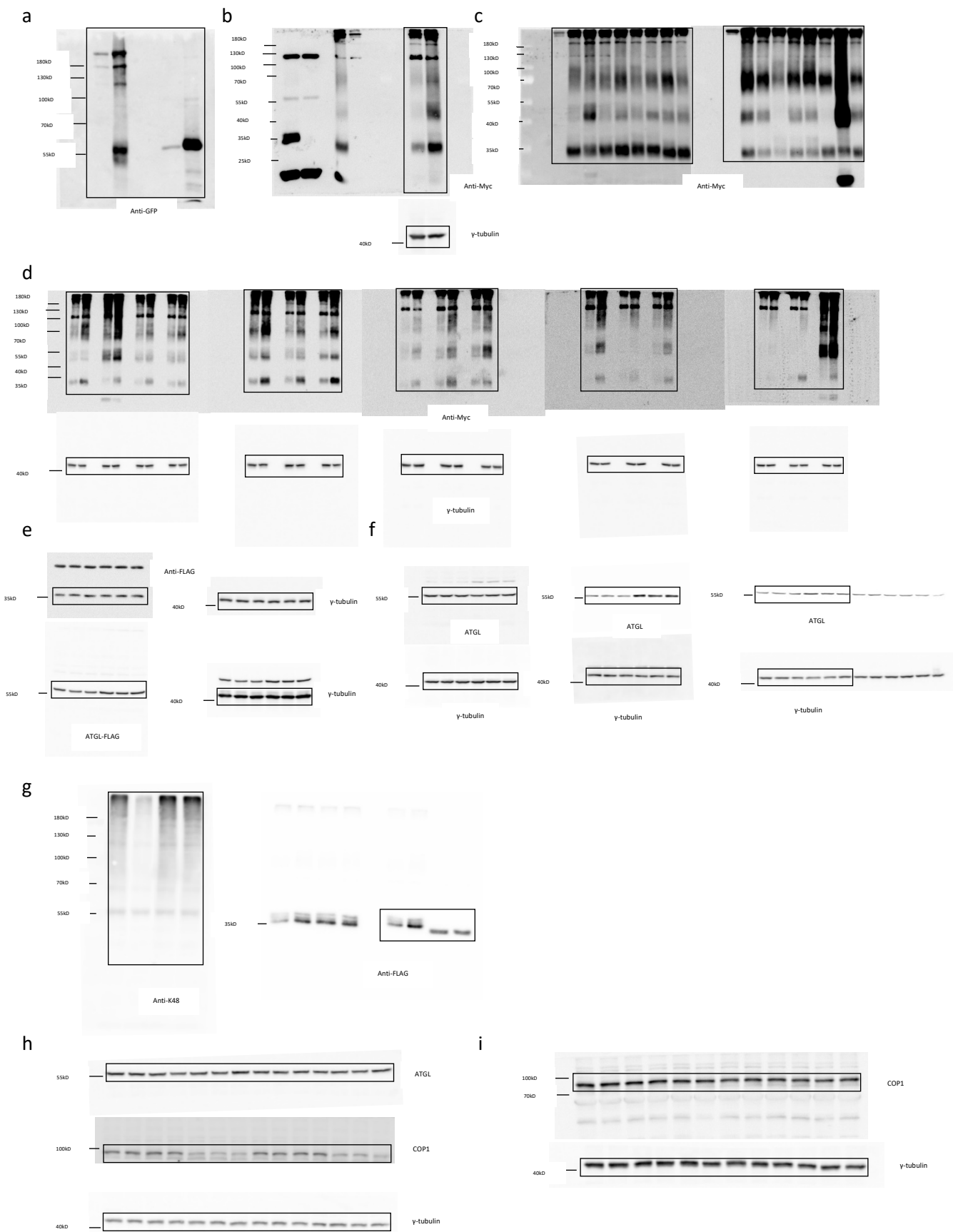

Supplement: Source Data Extended Data Fig. 6 — Uncropped western blot. [file 42255_2021_489_MOESM25_ESM.pdf]

Extended Data Fig.7

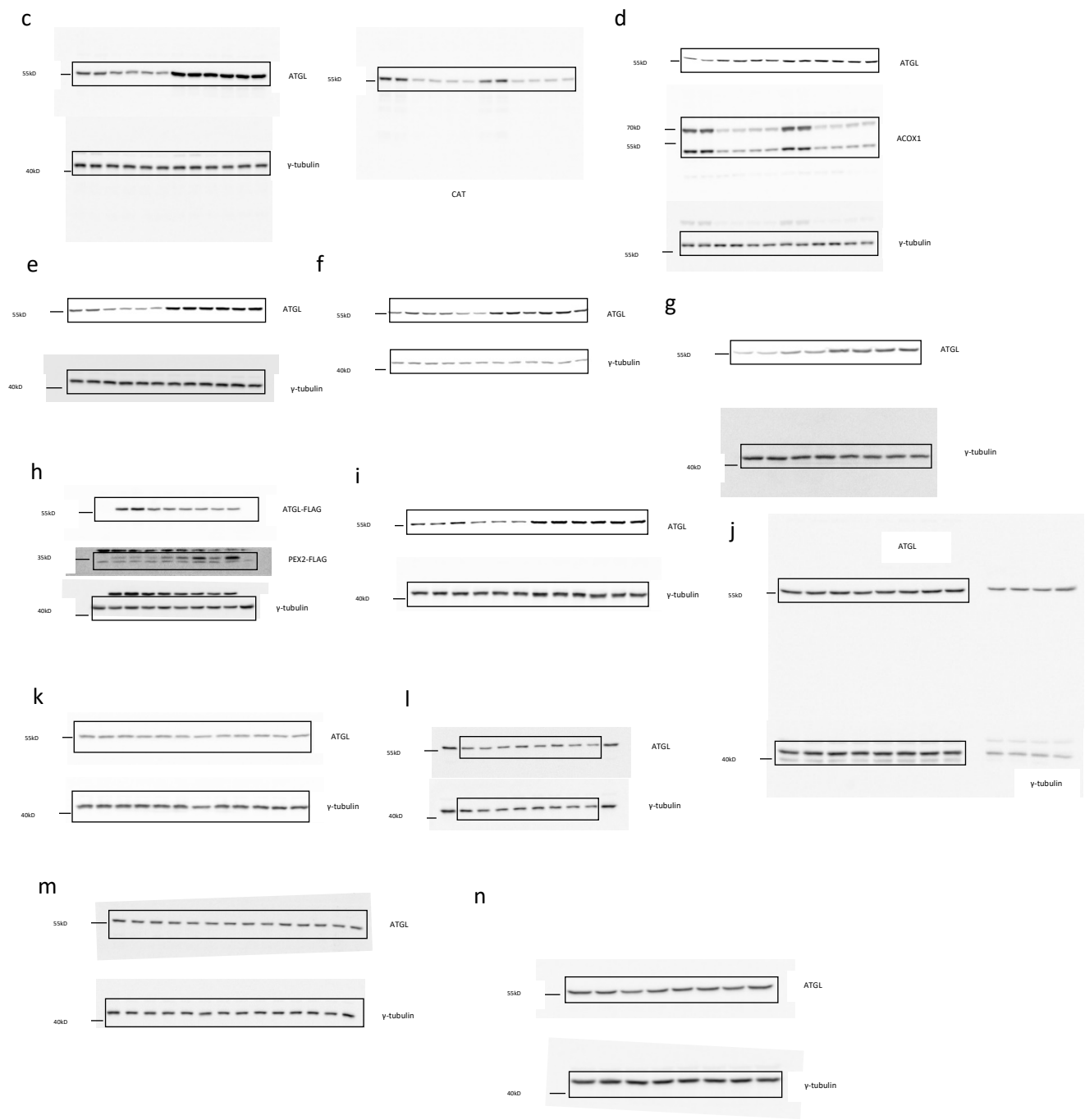

Supplement: Source Data Extended Data Fig. 7 — Uncropped western blot. [file 42255_2021_489_MOESM27_ESM.pdf]

Extended Data Fig.8

c

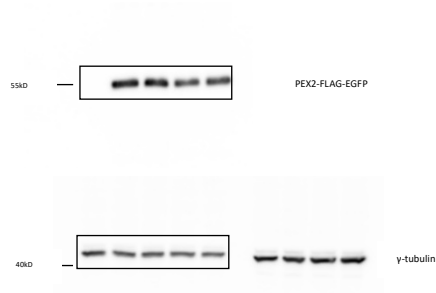

g

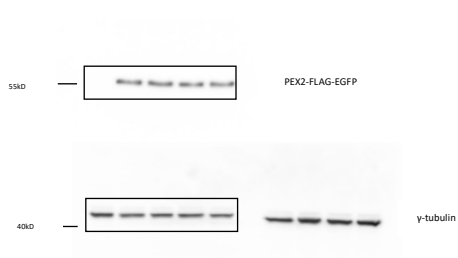

h

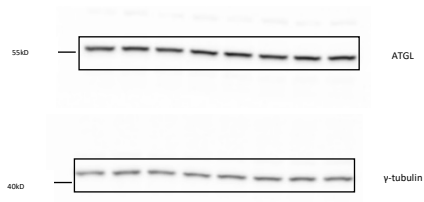

k

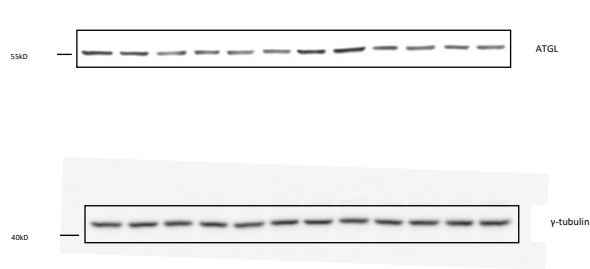

l

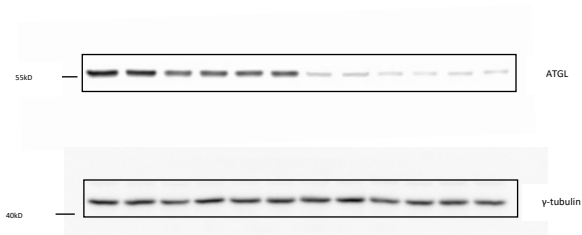

Supplement: Source Data Extended Data Fig. 8 — Uncropped western blot. [file 42255_2021_489_MOESM29_ESM.pdf]

Extended Data Fig.9

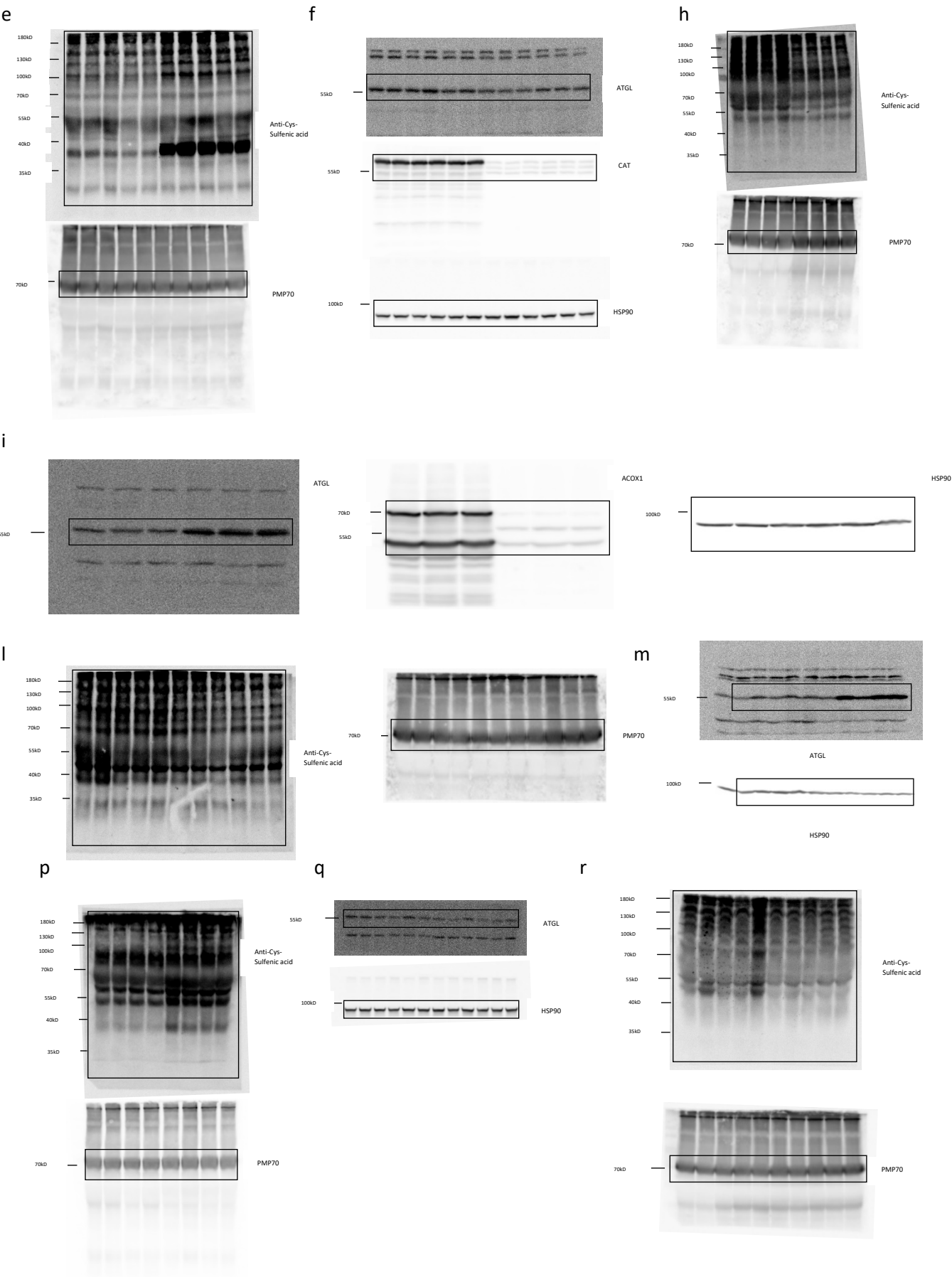

Supplement: Source Data Extended Data Fig. 9 — Uncropped western blot. [file 42255_2021_489_MOESM31_ESM.pdf]

Extended Data Fig.10

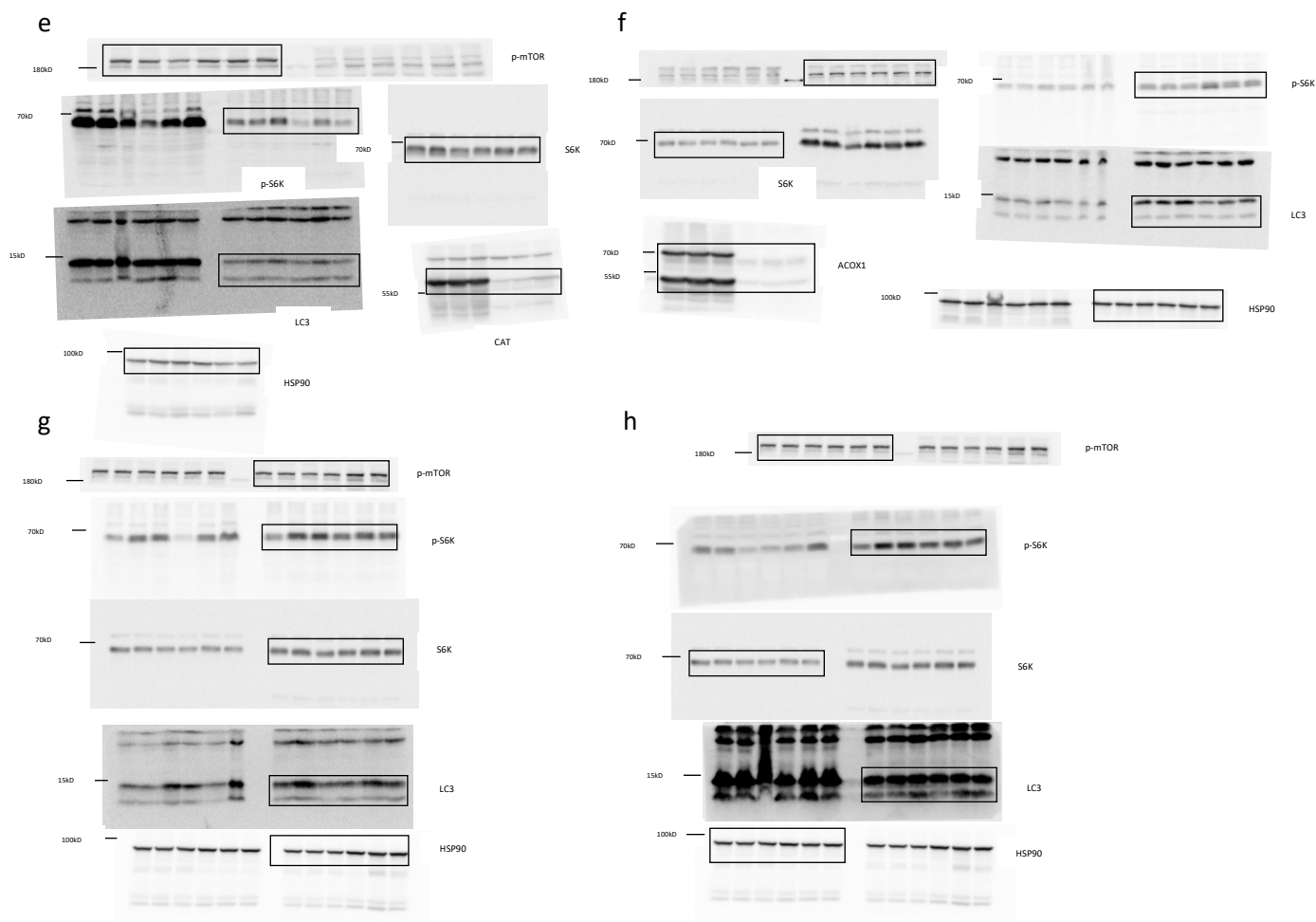

Supplement: Source Data Extended Data Fig. 10 — Uncropped western blot. [file 42255_2021_489_MOESM33_ESM.pdf]
